# Fibroid-like Uterine Lesions and Fibroid-Associated Structural Changes in a Long-Term Murine Hepatitis Virus-1 Model

**DOI:** 10.3390/microorganisms14102405

**Published:** 2026-10-20

**Authors:** Arumugam R. Jayakumar, Rajalakshmi Ramamoorthy, Urja C. Patel, Roberto Ruiz-Caordero, Dibe M. Di Gregorio, Neil Ferland, Smrati Sabnani, María Jose Araque, Gene Burkett, Michael J. Paidas

**Affiliations:** 1 Tamer Family Department of Obstetrics, Gynecology and Reproductive Sciences, Miller School of Medicine, University of Miami, Miami, FL 33136, USA; rxr1310@med.miami.edu (R.R.); ucp2@miami.edu (U.C.P.); dmdigrego@gmail.com (D.M.D.G.); smratigynaec93@gmail.com (S.S.); mxa5790@miami.edu (M.J.A.); gburkett@med.miami.edu (G.B.); 2 Pathology and Laboratory Medicine, Miller School of Medicine, University of Miami, Miami, FL 33136, USA; rxr1314@med.miami.edu; 3 School of Medicine, Universidad Central del Caribe, Bayamón, PR 00960, USA; 4 Department of Pediatric Infectious Disease, School of Medicine, University of Miami Miller, Miami, FL 33136, USA; nxf397@miami.edu; 5 Department of Biochemistry and Molecular Biology, School of Medicine, University of Miami Miller, Miami, FL 33136, USA

**Keywords:** fibroid, long COVID, menstrual bleeding, murine hepatitis virus-1, uterus

## Abstract

The number of patients with long COVID continues to rise as COVID-19 persists, yet research on its implications for female reproductive health remains scarce. Long COVID is linked to an increased risk of abnormal uterine bleeding, heavier menstrual flow, prolonged periods, and breakthrough bleeding. We therefore examined whether uterine changes occur in an animal model of long COVID. Female A/J mice were inoculated with murine hepatitis virus strain 1 (MHV-1), a murine coronavirus model used to investigate long COVID-like sequelae, and a subset of infected mice received the synthetic peptide SPIKENET (SPK). Mice infected with murine hepatitis virus strain 1 (MHV-1) showed chronic uterine inflammation and myometrial changes exhibiting fibroid-like features one year post-infection. We also observed changes in α-SMA, desmin, h-caldesmon, and vimentin staining, suggesting a myometrial origin for the lesions. Further, we found pathological changes that include hyperplastic foci in the outer layer of the myometrium, a uterus with intraluminal proteinaceous fluid, and a circumscribed, round-to-oval focus of benign hyperplastic myometrium with spindle nuclei and eosinophilic cytoplasm, with interspersed small, round lymphocytes and plasma cells, consistent with mild chronic inflammation. MHV-1-inoculated mice also showed an oviduct with several lymphocytes located toward the lumen, as well as a uterus with an ectatic lumen, inspissated intraluminal fibrinoid material, and mild-to-moderate subendometrial chronic inflammation (with scattered small lymphocytes). Additionally, immunofluorescence showed increased expression of conventional and unconventional uterine fibroid markers, including PLP1, versican, PR-A, PR-B, MSH2, TGF-β1 and 3, IGF-1, CA125, and HMGA2. Serum biomarker analysis showed increased IGF-1 levels but not increased levels of the other proteins identified by immunofluorescence. Furthermore, histopathology showed no uterine fibroid-like lesions in MHV-1-inoculated mice treated with the synthetic peptide SPK (known to prevent MHV-1 and SARS-CoV-2 infection in animals). Moreover, SPK prevented increases in the above-mentioned fibroid markers as measured by immunofluorescence and partially reduced IGF-1 expression in the serum. These findings suggest that uterine fibroids may develop during long COVID in humans.

## 1. Introduction

COVID-19 continues to pose a significant global public health burden, with ongoing SARS-CoV-2 transmission, morbidity, and mortality [1,2,3]. Despite global efforts to develop a COVID-19 vaccine to reduce mortality, there is no specific treatment for the viral infection [4,5,6]. The severity and the clinical, pathological, immunological, molecular, and functional changes observed in the murine hepatitis virus-1 (MHV-1, a beta coronavirus) mouse model recapitulate those observed in humans with SARS-CoV-2 [7,8,9,10,11,12,13,14,15,16,17,18]. MHV-1 infection of A/J mice results in a clinical SARS-like disease with high mortality. Pulmonary disease develops on days 2–3 post-MHV-1 infection, and severe lung injury occurs 6–7 days post-MHV-1 infection, reflecting a “severe” viral infection like that observed in humans with SARS-CoV-2 [7,8,9,10,11,12,13,14,15,16,17,18,19,20]. Results from MHV-1 can provide mechanistic insights into SARS-CoV-2 infection because both viruses are coronaviruses and share aspects of coronavirus biology, although they belong to different subgenera and infect different host species [7,8,9,10,11,12,13,14,15,16,17,18,19,20]. MHV-1 produces long-term neurological complications like those in humans with SARS-CoV-2 infection [7,8,9,10,11,12,13,14,15,16,17,18]. Clinical symptoms and death were not observed in mice inoculated with lower doses of MHV-1. Moreover, many of the changes identified in humans with SARS-CoV-2 infection, including hormonal and inflammatory events, are also observed in MHV-1-infected tissues [8,15,19,20,21].

While primarily recognized as a respiratory illness at the acute stage, major long COVID sequelae are chronic, multisystemic, and debilitating, primarily featuring profound fatigue, osteoporosis, subfertility, worsening diabetes, severe cognitive dysfunction, dysautonomia, and complications affecting various organ systems [4,22]. Other significant symptoms include post-exertional malaise, shortness of breath, cardiovascular issues, and sleep disturbances, with many patients developing chronic, autoimmune-like illnesses [22,23]. With the multitude of SARS-CoV-2 genetic mutations and the diverse range of post-infection complications, the exact pathophysiology remains elusive [4,22,24]. Emerging research suggests that long COVID can affect the menstrual cycle, ovarian health, and fertility [25,26].

Long COVID is thought to affect a broad spectrum of reproductive health conditions, primarily through persistent immune dysregulation, chronic inflammation, and hormonal imbalances. Reproductive health issues in women that are affected by long COVID include menstrual irregularities, worsening premenstrual symptoms, ovarian function and health, fertility challenges, pregnancy complications, sexual dysfunction, endometriosis, and related conditions [25,27,28,29]. These adverse events may arise from chronic inflammation and immune overactivation. Long COVID may be associated with abnormal uterine bleeding and possibly uterine fibroids (leiomyomas) in humans, but evidence for direct fibroid development is limited.

Uterine fibroids originate from myometrial smooth muscle cells and are most commonly caused by mutations in the genes *MED12* (50–85% of cases) and *HMGA2/1* (~10% of cases) [30,31]. It has been reported that about 70–80% of women are affected during their lifetimes, making them the most common myometrial tumors [32]. Of those affected, up to 50% experience symptoms such as heavy menstrual bleeding, anemia, pelvic pressure, and pain [32,33]. The diagnosis of fibroids is frequently delayed, as diagnosis takes >5 years for one-third of patients, which causes increased morbidity and decreased quality of life, and is the leading cause of hysterectomies [32,34]. Two crucial menstrual cycle hormones, estrogen and progesterone, play key roles in fibroid development and growth, with progesterone activating ligands and cytokines in the WNT/β-catenin signaling pathway that support tumor stem cell proliferation [32,35]. The International Federation of Gynecology and Obstetrics (FIGO) classifies them by anatomic location: submucosal (types 0–2), intramural (types 3–5), subserosal (types 6–7), and cervical (type 8) [31]. These leiomyomas are common benign tumors that, when they grow, can lead to chronic, heavy menstrual bleeding, severe anemia, pelvic pressure/pain, infertility, and pregnancy complications such as miscarriages or preterm labor. Menorrhagia caused by fibroids can lead to severe, chronic anemia, which, in extreme, untreated cases, can be life-threatening. Furthermore, massive fibroids can obstruct the bowel or urinary tract, leading to kidney failure or other complications.

In this study of MHV-1-infected mice, we investigated the association between Long COVID and uterine fibroid incidence by examining the expression of proteolipid protein 1 (PLP1), versican, Progesterone Receptor A (PR-A), Progesterone Receptor B (PR-B), MSH2 (MutS homolog 2), TGF-β 1 and 3, IGF-1 (insulin-like growth factor-1), CA125, and HMGA2. We also explored the effect of SPIKENET (SPK), a 15-amino-acid synthetic peptide, on preventing fibroid-like growth. Our study, for the first time, shows a possible connection between Long COVID and fibroid-like lesions following MHV-1 infection in murine models. Thus, the primary objective of this study was to establish an initial in vivo proof of concept demonstrating that viral presence correlates with key histopathological and immunofluorescent hallmarks of fibroid development (e.g., smooth muscle cell proliferation and extracellular matrix deposition). Characterizing this descriptive phenotype is a necessary first step before accurately isolating downstream signaling mechanisms. This is highly significant because normal, healthy mice do not develop uterine fibroids (leiomyomas) on their own.

## 2. Materials and Methods

### 2.1. Mice

Female A/J mice (8 weeks of age, weighing 22 g) were purchased from Jackson Laboratories (Bar Harbor, ME, USA) and maintained in micro-isolated cages in the animal colony at the Biomedical Research Building animal isolation facility at the University of Miami Miller School of Medicine. They were fed a standard lab chow diet [Envigo 2918 irradiated (Teklad diet, Dublin, VA, USA)] and provided autoclaved tap water ad libitum. The study was conducted in accordance with the guidelines of the University of Miami Institutional Animal Care and Use Committee (IACUC protocol number 23–162, renewal approved on 12 September 2023).

### 2.2. Viral Inoculation and Experimental Group

MHV-1 was purchased from the American Type Culture Collection (ATCC, cat# VR 261, Manassas, VA, USA). Mice were split into (1) a healthy control group, (2) an MHV-1 virus-inoculated group, and (3) an MHV-1 virus-inoculated group that was treated with SPK. MHV-1 viral inoculation was performed as previously described [10,11,12,13,14,15,16]. Briefly, we inoculated groups 2 and 3 intranasally with 5000 PFU of MHV-1 and observed them to ensure adequate viral inhalation. MHV-1-inoculated mice (group 3) were additionally treated with 5 mg/kg body weight of SPK, as previously described by us [10,11,12,13,14,15,16].

While we used a larger sample size in the chronic cohort (the study began with different sample sizes: n = 6 for control, n = 10 for MHV-1, and n = 10 for MHV-1 + SPK), during the one-year study period, some animals were removed from the study and euthanized due to illness, resulting in fewer animals in each group after one year of MHV-1 infection. The smaller sample size for ELISA testing (see below) also reflects low serum viability, likely due to reduced blood volume related to age and illness, infection-associated dehydration and weight loss, impaired cardiovascular perfusion near terminal disease, and potentially increased coagulation. We previously reported an interesting finding [10]: in acute infection, the heart is less severely affected than other organs such as the lung, brain, liver, and kidney, whereas in long-term post-infection, the heart is severely affected [10]. This is a limitation of our study, and future research should validate these findings in larger cohort studies.

### 2.3. Uterus Collection and Histological Analysis

We performed a careful histological analysis after MHV-1 infection. Briefly, we removed the uterus one year post-infection; for histopathologic correlation, we fixed uterine sections from normal and infected mice in 10% formalin for 24 h, processed them routinely for paraffin sections, and stained them with Hematoxylin and Eosin (H&E). Uterine fibroid-like lesions were identified by distinctive architecture and cellularity (whorled fascicles, spindle cells, borders, and cellularity), cellular and nuclear characteristics (cytoplasm, nuclei, atypia, and mitosis), stroma and extracellular matrix (collagen bundles, hyalinization), vascularity (thick-walled vessels), and common degenerative changes such as hyaline degeneration, red degeneration (ischemic), myxoid degeneration, and calcification. Although leiomyomas are benign, H&E can distinguish them from leiomyosarcomas (malignant) by coagulative tumor necrosis, significant nuclear atypia, and a high mitotic count. We used immunofluorescence to analyze factors implicated in general fibroid progression.

### 2.4. Immunofluorescence

Paraffin-embedded tissue sections from the healthy control and MHV-1-infected groups (10 microns) were incubated with specific antibodies against CA125 (cat#60261-1-Ig monoclonal antibody, Proteintech, Rosemont, IL, USA); MSH2 (cat#A1121 polyclonal antibody, ABclonal, Woburn, MA, USA); TGF-Beta (cat#MAB2401 monoclonal antibody, R&D systems, Minneapolis, MN, USA); nucleocapsid protein (cat#MA5-29981 monoclonal antibody, Invitrogen, Kingwood, TX, USA); S1 (cat#GTX635671 monoclonal antibody, Genetex, Irvine, CA, USA); HMGA2 (cat#20795-1-ap polyclonal antibody, Proteintech, Rosemont, IL, USA); PR-A (cat#A22061 monoclonal antibody, ABclonal, Woburn, MA, USA); PR-B (cat#3157T monoclonal antibody, Cell signaling, Danvers, MA, USA); ESR (cat#A2546 polyclonal antibody, ABclonal, Woburn, MA, USA); versican (cat#A19655 monoclonal antibody, ABclonal, Woburn, MA, USA); PLP1 (cat#A20009 polyclonal antibody, ABclonal, Woburn, MA, USA); and IGF-1 (cat#A0303 polyclonal antibody, ABclonal, Woburn, MA, USA). All were used at a 1:300 dilution. The sections were then incubated with the respective secondary antibodies (Alexa Fluor 488 or Alexa Fluor 546 goat anti-mouse or rabbit IgG (Life Technologies, San Diego, CA, USA)) at a concentration of 1:400. Fluorescent images were randomly captured using a Leica Thunder imager (Leica Microsystems Inc., Deerfield, IL, USA). The images were randomly collected in a “blinded” manner. We captured at least 12 fluorescent images and quantified them using Volocity 6.0 High-Performance Cellular Imaging Software (PerkinElmer, Waltham, MA, USA), as described previously [36].

### 2.5. Enzyme-Linked Immunosorbent Assay (ELISA)

We obtained serum samples from blood and stored them at −20 °C. The levels of Versican (Cat no# ab279409, Abcam, Waltham, MA, USA), IGF1 (Cat no# ab100695, Abcam, Waltham, MA, USA), TGF-β (Cat no# ab119577, Abcam, Waltham, MA, USA), PLP1 (Cat no# MBS261968, Mybiosource, San Diego, CA, USA), Progesterone ELISA kit (cat# K025-H1, Arbor Assays, Ann Arbor, MI, USA) and 17 beta Estradiol (17 beta Estradiol ELISA kit, Cat# Ab108667 (Abcam, Waltham, MA, USA)) were quantified using specific ELISA kits following the manufacturer’s instructions, including the creation of standard curves. We determined concentrations based on these standard curves.

### 2.6. Real-Time PCR

The sequences of primers used were based on a published study [37]. We used 2 μg of RNA per sample for cDNA synthesis, following the manufacturer’s protocol with a high-capacity cDNA reverse transcription kit (Cat #4374967, Thermo Fisher Scientific, Waltham, MA, USA). We selected MHV-1 primer sequences (Table 1, [37]) and purchased them from Oligos (Sigma-Aldrich, Burlington, MA, USA). We performed quantitative real-time PCR using an iCycler (Bio-Rad Laboratories, Hercules, CA, USA) and determined the cycle threshold (Ct) with iCycler software version 3. All reactions were done in triplicate, and Ct values were normalized to the average Ct of GAPDH.

### 2.7. Statistical Analysis

All ELISA data were analyzed using GraphPad Prism version 9.5.1. One-way ANOVA was used to assess significant differences between samples (* = *p* ≤ 0.05; ** = *p* ≤ 0.01 was considered significant; ns = non-significant).

## 3. Results

### 3.1. Histological Analysis and IHC Markers

**Figure 1:**
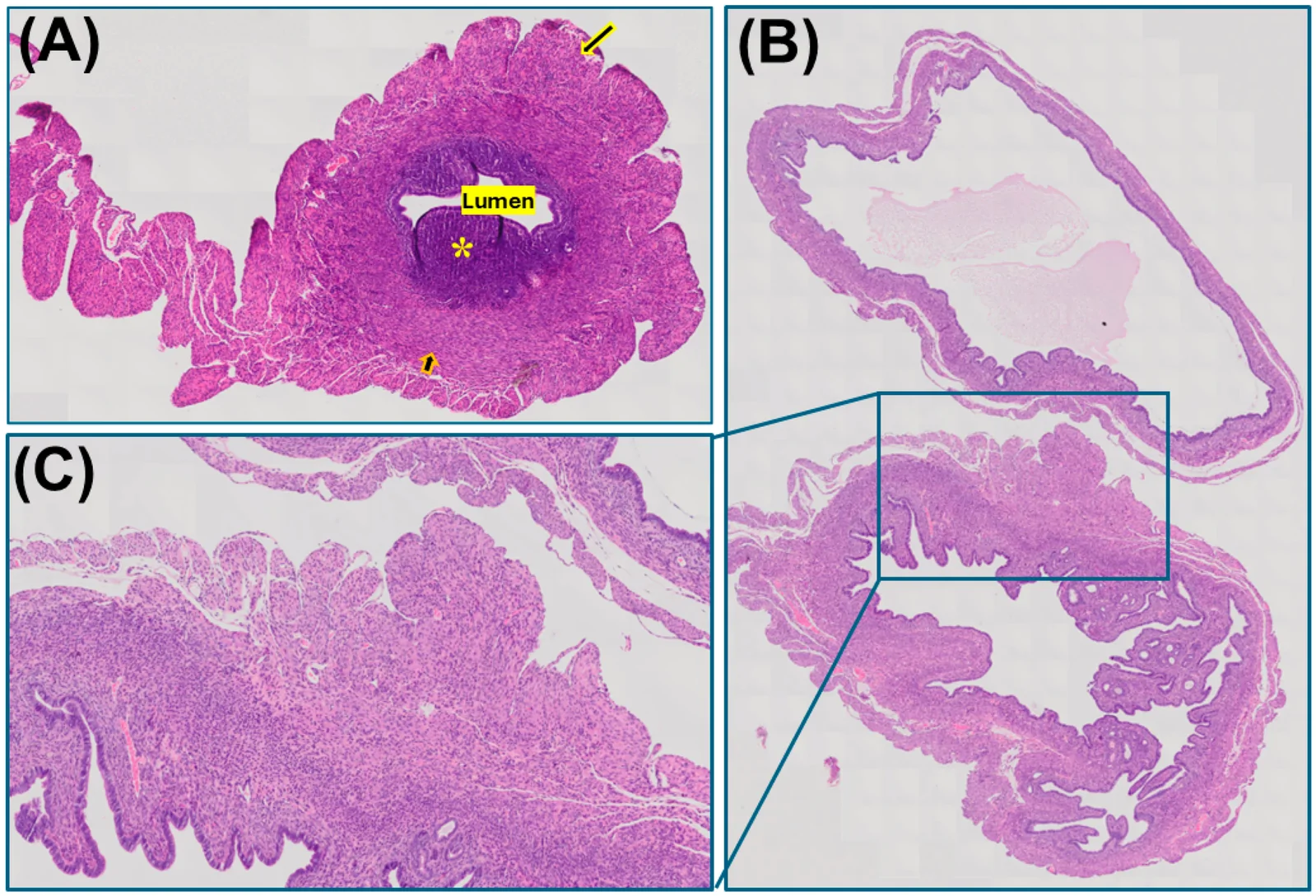
(**A**) Transverse section of a uterus from a normal mouse showing endometrium (asterisk), inner myometrium (short arrow), outer myometrium (long arrow), and serosa with no significant pathological abnormalities. (**B**,**C**) Two cross-sections of different segments of the uterus. The inset shows a hyperplastic focus in the outer myometrial layer (H&E) (n = 6 for control and n = 3 for MHV-1). Original magnification 66×.

Our pilot studies show notable changes in the uterus, mainly the presence of fibroid-like lesions. A transverse section of the uterus from a normal mouse shows no significant histological changes in the endometrium, inner myometrium, outer myometrium, and serosa, with no significant pathological abnormalities (Figure 1A).

**Figure 2:**
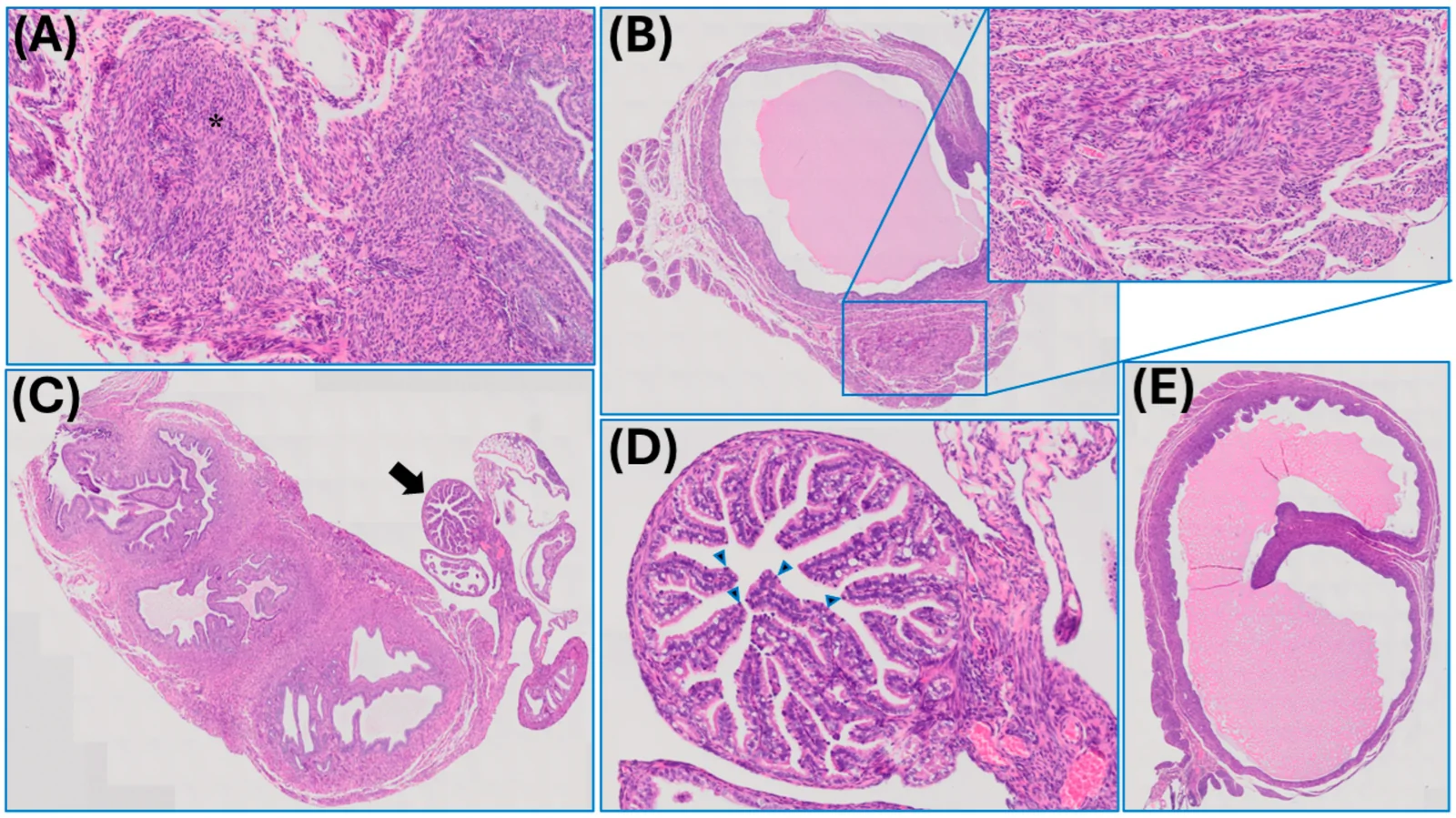
(**A**) A cross-section of the uterus shows a hyperplastic nest of smooth muscle fibers with architectural disarray in the inner myometrium (asterisk). (**B**) Cross-section of the uterus with intraluminal proteinaceous fluid and a circumscribed, round-to-oval focus of benign hyperplastic myometrium with spindle nuclei and eosinophilic cytoplasm with interspersed small, round lymphocytes and plasma cells, consistent with mild chronic inflammation (inset). (**C**) Cross-section of the distal uterine horn with attached oviduct (arrow) and fimbriae, showing several foci of chronic inflammation, including lymphoepithelial lesions and lymphocytes located toward the lumen. (**D**) Cross-section of the oviduct with several lymphocytes located toward the lumen (arrowheads). (**E**) Cross-section of a uterus with an ectatic lumen and inspissated intraluminal fibrinoid material (n = 6 for control and n = 3 for MHV-1). Original magnification 66×.

However, mice inoculated with MHV-1 exhibit various uterine changes one year post-infection. These include a hyperplastic focus in the outer layer of the myometrium (Figure 1B,C); a hyperplastic nest of smooth muscle fibers with architectural disarray in the inner myometrium (Figure 2A); a uterus with intraluminal proteinaceous fluid and a circumscribed, round-to-oval focus of benign hyperplastic myometrium with spindle nuclei and eosinophilic cytoplasm with interspersed small, round lymphocytes and plasma cells, consistent with mild chronic inflammation (Figure 2B); and the presence of a distal uterine horn with attached oviduct and fimbriae and with several foci of chronic inflammation, including lymphoepithelial lesions and lymphocytes located towards the lumen (Figure 2C). MHV-1-infected mice (one year post-infection) also show an oviduct with several lymphocytes located towards the lumen (Figure 2D), as well as a uterus with an ectatic lumen and inspissated intraluminal fibrinoid material (Figure 2E).

**Figure 3:**
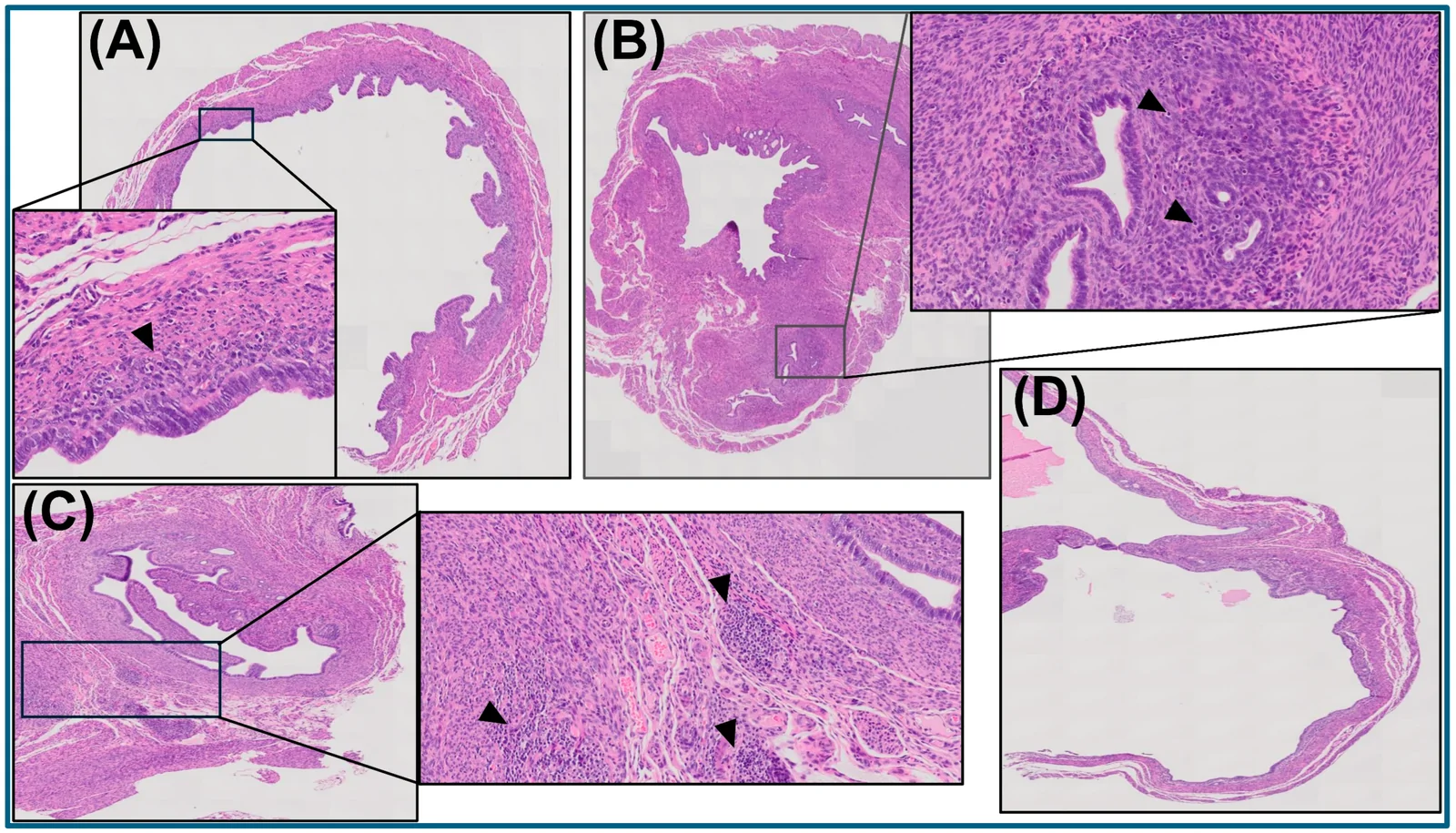
(**A**) Partial cross-section of the uterus with mild mixed subepithelial inflammation (inset, arrowhead). (**B**) Cross-section of a uterus with mild-to-moderate sub-endometrial chronic inflammation (inset, arrowheads highlight scattered small lymphocytes). (**C**) Cross-section of a uterus with interstitial foci of chronic inflammation, including small, mature-appearing lymphocytes and plasma cells (arrowheads, inset). (**D**) Cross-section of a uterus with an ectatic lumen and inspissated intraluminal fibrinoid material. (n = 6 for control and n = 3 for MHV-1). H&E, original magnification 66×.

A partial cross-section of a uterus with mild mixed subepithelial inflammation (Figure 3A), uterus with mild-to-moderate sub-endometrial chronic inflammation (with scattered small lymphocytes) (Figure 3B), uterus with interstitial foci of chronic inflammation, including small, mature-appearing lymphocytes and plasma cells (Figure 3C), and frequent inspissated intraluminal fibrinoid material (Figure 3D) were identified long-term post-MHV-1 infection. These preliminary findings suggest a possible association between long COVID and uterine fibroid-like lesion development.

**Figure 4:**
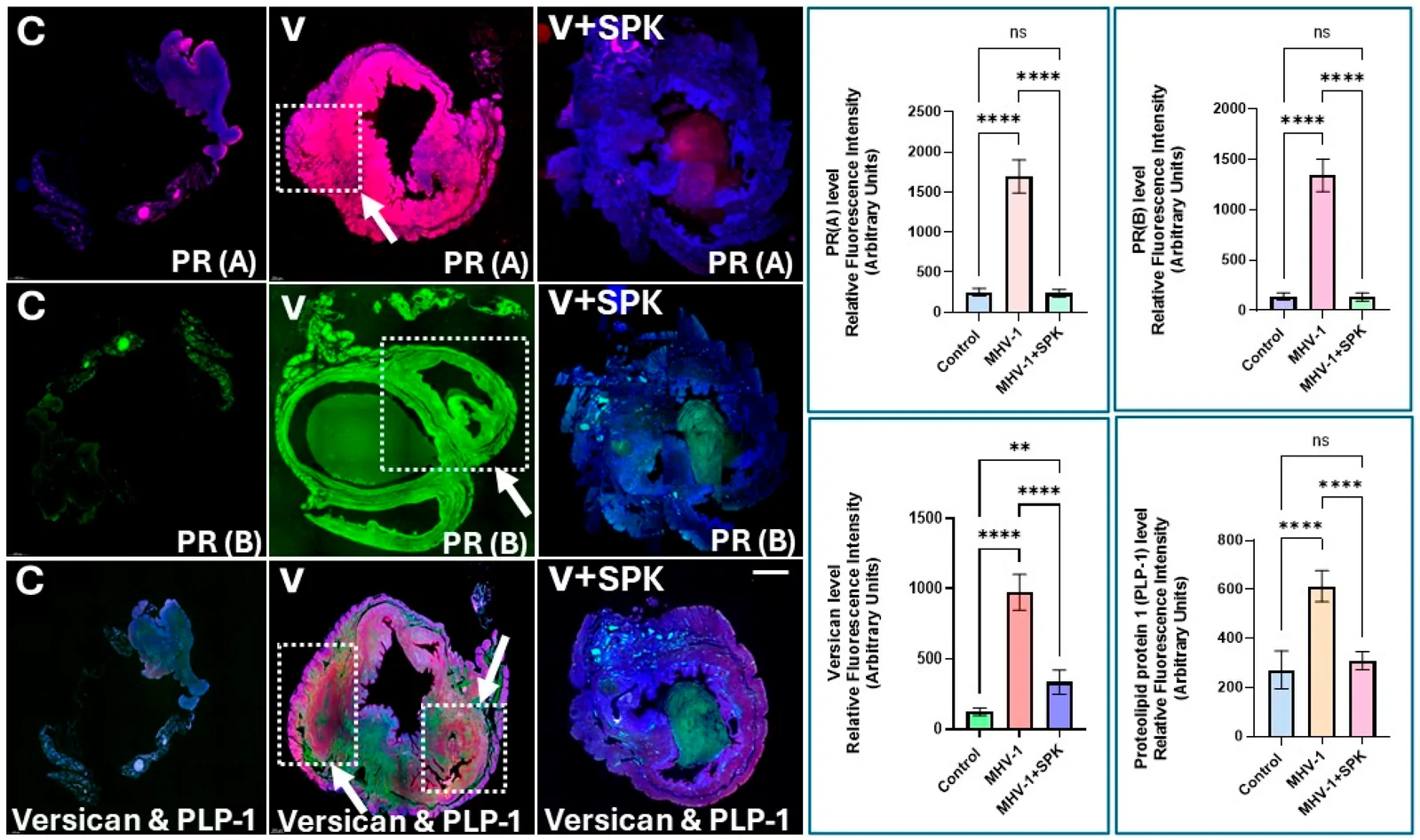
Representative immunofluorescence images of the uterus from MHV-1-infected mice (one year post-infection) showed increased levels of various factors commonly reported in uterine fibroids. These include Progesterone Receptor A (PR-A), Progesterone Receptor B (PR-B), Versican, and Proteolipid Protein 1 (PLP-1) (n = 5). The presence of fibroid-like lesions is demarcated by the segmented boxes and arrows. Treatment of these mice with SPIKENET (SPK) [11] prevented increases in these factors and fibroid-like lesions (n = 6 for control, n = 5 for MHV-1, and n = 5 for MHV-1+SPK). ** *p* < 0.01 and **** *p* < 0.0001. Scale bar = 500–600 µm. C, control; V, virus; ns = non-significant.

**Figure 5:**
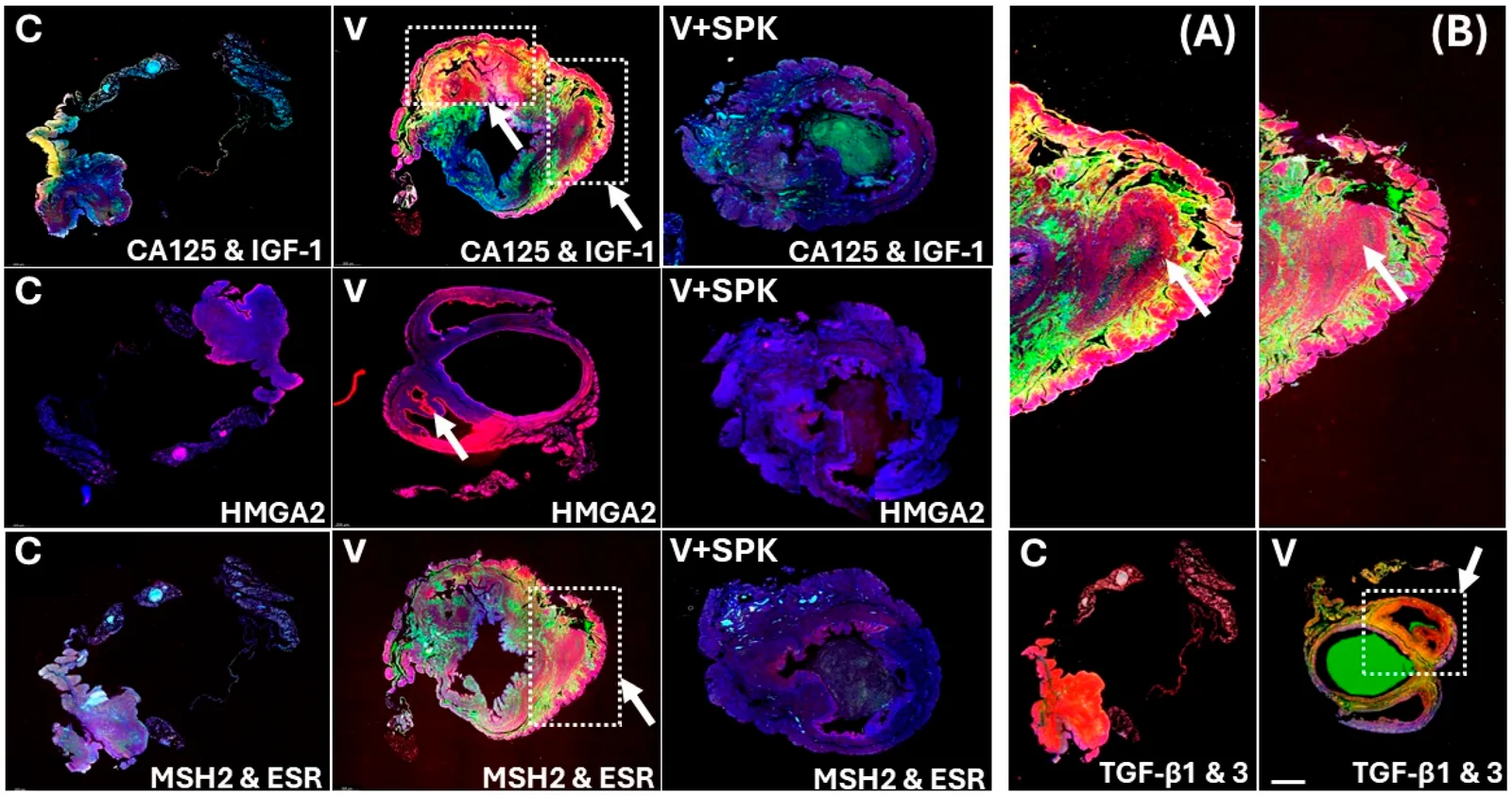
Representative immunofluorescence images of the uterus from MHV-1-infected mice (one year post-infection). Semiquantitative analysis of these images showed increased levels of various factors in MHV-1-infected mice that are commonly reported in uterine fibroids (see Figure 7 below for quantitation). These include CA125, IGF-1, HMGA2, MSH2, ER-α (ESR), and TGF-β1 and TGF-β3 (n = 5). The presence of a fibroid-like lesion is demarcated by the segmented boxes and short white arrows. (**A**) Enlarged image of the fibroid-like lesion shows increased levels of IGF-1 receptor (IGF-1, red) and CA125 (green) in the uterus of virus-infected mice. IGF-1r is highly expressed (long white arrow). (**B**) Enlarged image of fibroid-like lesions shows ER-α (green) and MSH2 (red) in the virus-infected mouse uterus. ER-α is more highly expressed than MSH2 (long white arrows). Treatment of these mice with SPK prevented increases in these factors and fibroid-like lesions (n = 6 for control, n = 5 for MHV-1, and n = 5 for MHV-1 + SPK). Scale bar = 500–600 µm. C, control; V, virus.

Immunofluorescence imaging of the uterus from MHV-1-infected mice revealed increased levels of various factors generally reported in uterine fibroids. These include proteolipid protein 1 (PLP1) (Figure 4), which is overexpressed in uterine fibroids due to gene hypomethylation; versican, a large chondroitin sulfate proteoglycan found in the extracellular matrix (ECM) of the uterus (Figure 4); Progesterone Receptor A (PR-A), an isoform of the progesterone receptor, along with PR-B, both of which are overexpressed in uterine fibroids compared with the normal mouse uterus; and MSH2 (MutS homolog 2) (Figure 5), a DNA mismatch repair gene, whose expression is downregulated. Some studies have observed elevated protein levels in uterine fibroids. In preliminary studies, we found increased MSH2 protein levels in the uteri of MHV-1-infected mice (Figure 5).

**Figure 6:**
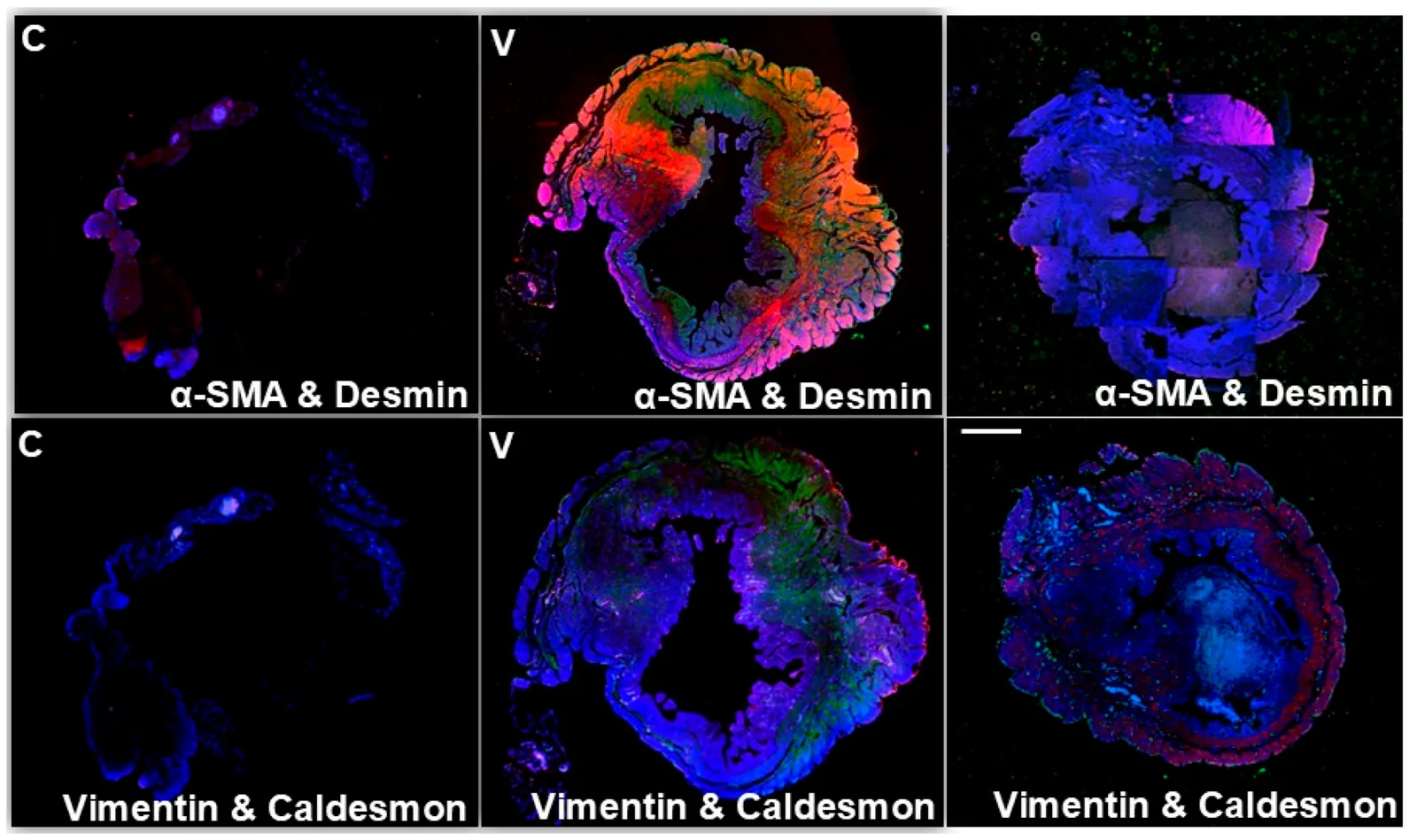
Representative immunofluorescence images of the uterus from MHV-1-infected mice (one year post-infection). Semiquantitative analysis of these images showed increased levels of various factors in MHV-1-infected mice that are commonly reported in uterine fibroids. These include α-SMA, desmin, vimentin, and caldesmon (n = 6 for control, n = 5 for MHV-1, and n = 5 for MHV-1 + SPK). Further, treatment of these mice with SPK prevented increases in these factors and fibroid-like lesions. Scale bar = 500–600 µm. C, control; V, virus.

**Figure 7:**
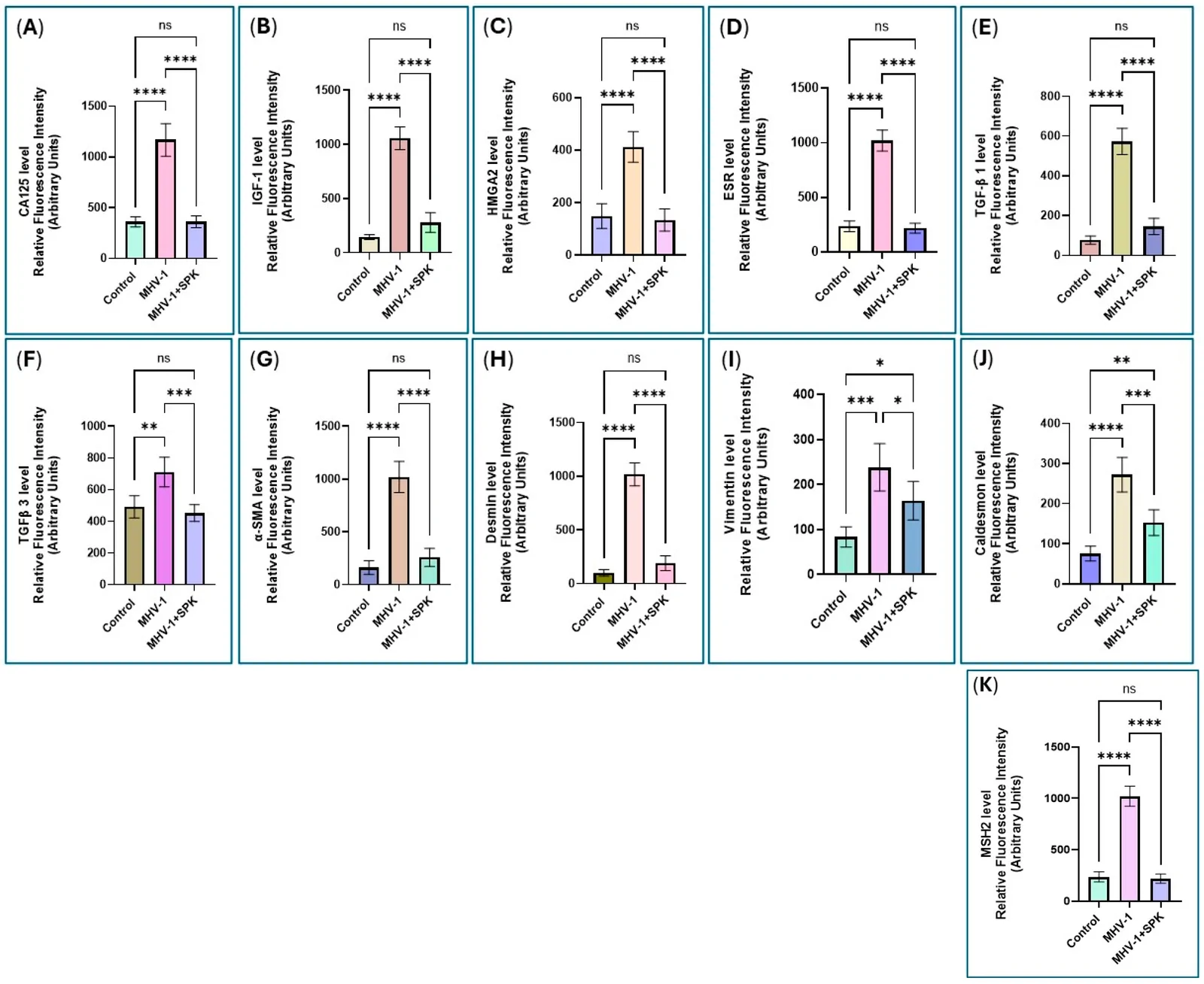
Semiquantitative analysis of immunofluorescence images in Figure 5 and Figure 6. * *p* < 0.05, ** *p* < 0.01, *** *p* < 0.001, and **** *p* < 0.0001. ns = non-significant.

Estrogen receptors (ER-α and ER-β) are generally overexpressed in uterine fibroids (leiomyomas) compared with normal uterine muscle fibers. We observed a similar trend in the uterus of MHV-1-infected mice (Figure 5). Additionally, we detected alpha-SMA, desmin, vimentin, and caldesmon in uterine fibroid-like lesions from MHV-1-infected mice (Figure 6, quantification in Figure 7). Further, these same factors were significantly reduced in MHV-1-infected mice that were treated with SPK (Figure 6; quantification in Figure 7).

While physical, architectural, and functional characterization (such as volumetric growth tracking or tissue mechanics) may provide more comprehensive validation of fibroid development than molecular markers alone, our current study aimed to establish baseline histopathological and immunofluorescence profiles of these lesions, demonstrating cellular changes and localized extracellular matrix deposition. Our reliance on histochemistry and immunofluorescence provides a static snapshot rather than a dynamic, functional view of development or defining the precise molecular pathway or causal biochemical mechanisms by which this virus drives fibroid formation. Thus, future studies will build on these profiles by integrating longitudinal micro-ultrasound tracking with biomechanical stiffness testing to fully characterize the physical progression of the fibroid phenotype.

**Figure 8:**
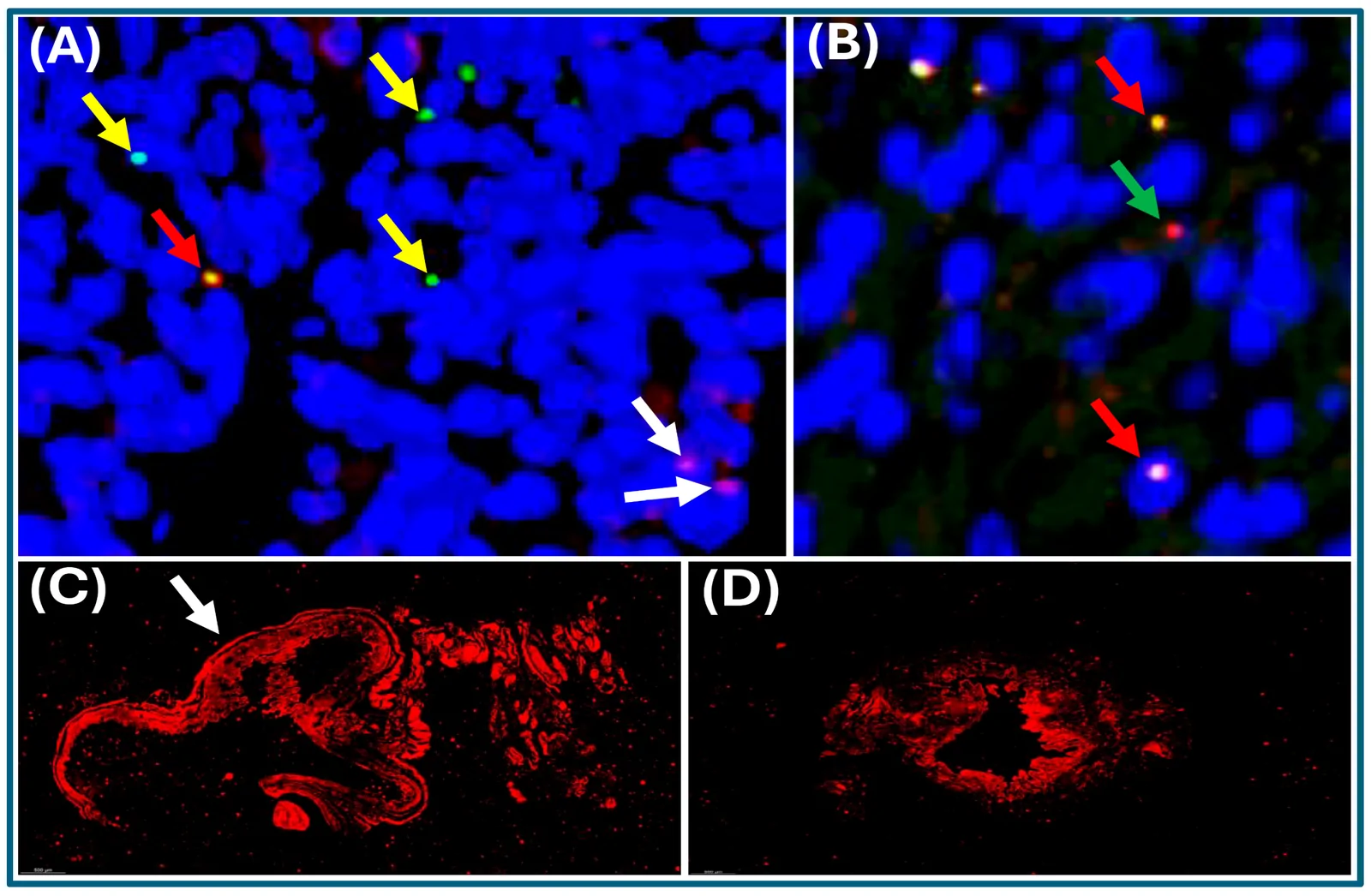
Immunofluorescence images of the uterus from MHV-1-infected mice (1 year post-infection) reveal the presence of the virus and viral particles. (**A**) Both the virus and viral particles are present (green with yellow arrows, S1 protein; red with white arrows, nucleocapsid protein; and orange with a red arrow, the virus). (**B**) Red arrows indicate the virus, and the green arrow indicates the nucleocapsid protein. (**C**) CEACAM1 level in the uterus of control mice (white arrow). (**D**) CEACAM1 level in the uterus of MHV-1-infected mice. CEACAM1 levels are reduced in uterine tissue from MHV-1-infected mice (n = 6 for control, n = 5 for MHV-1), similar to the reduction in ACE2 levels in humans with COVID-19, in both acute and long-term post-infection.

We also found increased levels of IGF-1 (insulin-like growth factor-1) (IGF-1 and its receptor IGF-1R are significantly increased in uterine fibroid-like lesions compared to normal myometrial tissue), CA125 (CA125 levels can increase in the presence of uterine fibroids, particularly when they are large, although they are not specific to fibroids), and HMGA2 (high-mobility group AT-hook 2), which is significantly increased/overexpressed in uterine fibroids (Figure 5). We also found increased levels of serum IGF-1 and fibroid IGF-1R, as well as TGF-beta 1 and 3 levels in these fibroid-like lesions (Figure 5). Notably, we observed the presence of the virus and virus particles, as well as a reduction in the receptor for mouse hepatitis virus (MHV), carcinoembryonic antigen-related cell adhesion molecule 1 (CEACAM1), throughout the entire uterus and in the fibroid-like lesions (Figure 8).

The presence of the virus, as well as viral particles, is higher in the fibroid-like lesions compared to the surrounding normal tissues (see the analysis section for interpretation). In contrast, SPK treatment reversed the expression of most genes, thereby restoring the gene profiles to those observed in control mice.

### 3.2. Serum Biomarkers

**Figure 9:**
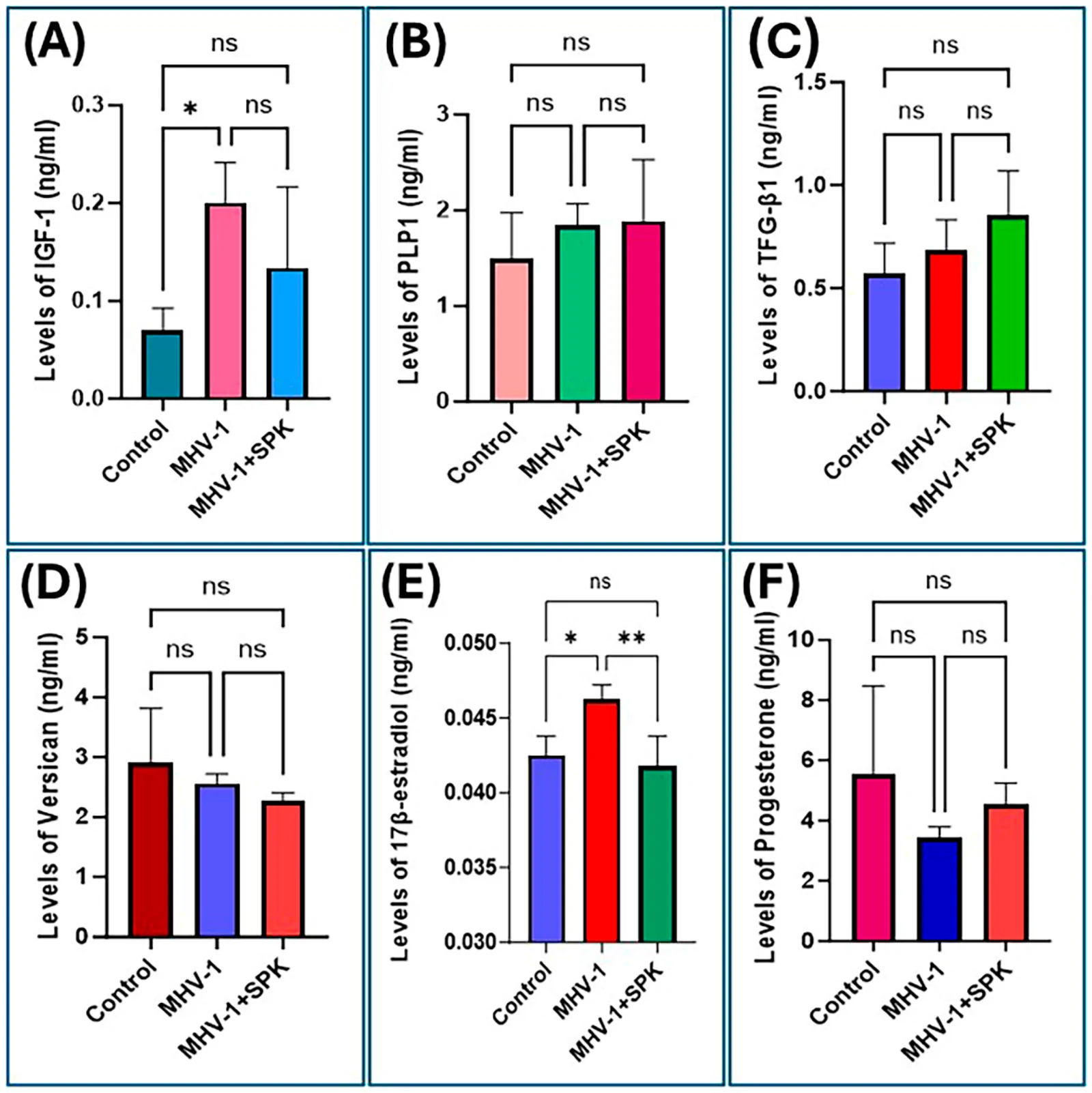
Serum markers and hormone levels in the control, MHV-1, and MHV-1+SPK groups. (**A**) MHV-1 significantly increased serum IGF-1 levels in MHV-1-inoculated mice (one year post-infection). (**B**,**C**) PLP-1 and TGF-β1 levels also increased but were not significantly different from controls. Furthermore, Versican levels decreased in MHV-1-inoculated mice (**D**), whereas 17β-estradiol was significantly increased in MHV-1-infected mice (**E**). Progesterone levels decreased but were not significantly different from controls (**F**). Data are presented as mean ± standard error; n = 3 per group. Statistical analyses were performed using one-way ANOVA followed by Tukey’s multiple-comparisons test (n = 6 for control, n = 3 for MHV-1, and n = 3 for MHV-1 + SPK). * *p* < 0.05 versus control; ** *p* < 0.01 versus the MHV-1 + SPK group; ns, not statistically significant from respective groups.

We further evaluated circulating biomarkers associated with uterine fibroid development. We quantified serum IGF-1, PLP-1, TGF-β1, and versican levels by ELISA (Figure 9). MHV-1-inoculated mice exhibited a significant increase (2.5-fold) in serum IGF-1 levels compared with control mice one year post-infection (Figure 9A). PLP-1 and TGF-β1 also increased in serum in MHV-1-inoculated mice relative to controls; however, these differences did not reach statistical significance (Figure 9B,C). In contrast, circulating versican levels were reduced in the MHV-1-inoculated mice compared with controls (Figure 9D). SPK treatment produced variable effects across biomarkers, with trends toward normalization in several serum protein levels, though these changes were not statistically significant.

We have also measured additional critical markers and found increased levels of 17-β estradiol but not progesterone in the serum of MHV-1-infected mice (Figure 9E,F). This effect (17-β estradiol elevation) was normalized in SPK-treated mice (Figure 9E). Notably, progesterone levels are not altered in the serum of MHV-1-infected mice (Figure 9F), likely because infection-related stress and inflammation may suppress the hypothalamic–pituitary–ovarian axis, reducing systemic progesterone while local inflammatory pathways sustain 17-β estradiol (E2) signaling and fibroid-like changes. Although elevated levels of IGF-1, TGF-β, hormone receptors, PRa, PRb, CA125, MSH2, versican, PLP-1, HMGA2, extracellular matrix markers, and serum and tissue 17-β estradiol were detected, suggesting the presence of fibroid-like lesions or fibroid formation, these findings should not be taken as direct evidence of increased cellular proliferation or tumor development without further functional validation.

### 3.3. MHV-1 RNA in Uterine Tissues

**Figure 10:**
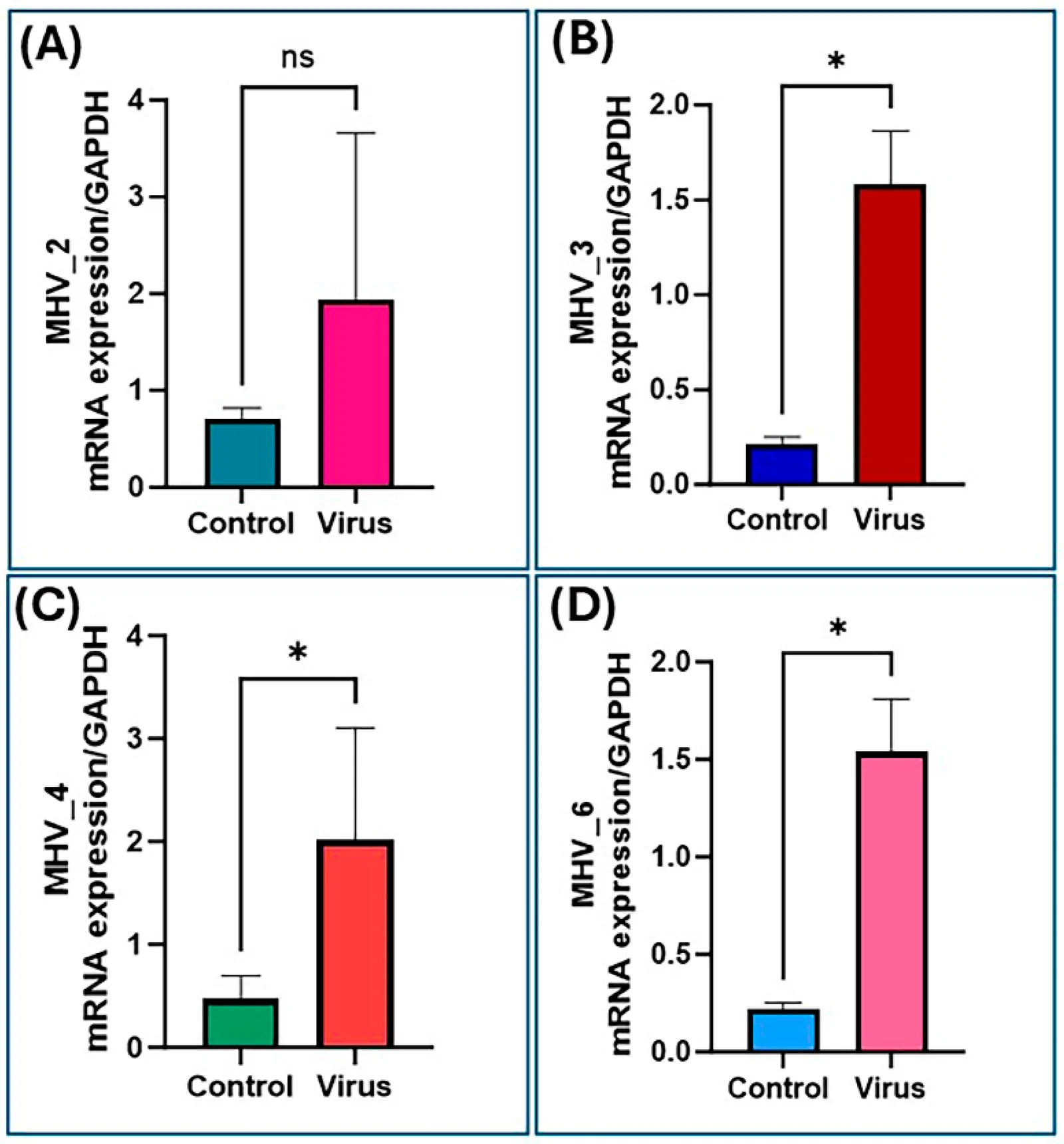
RT-PCR analysis of MHV RNA expression in tissues from both control and MHV-1-inoculated mice. We assessed MHV RNA levels using six primers (see Table 1). Primer (**A**) shows increased MHV-2 mRNA expression, but the change is not statistically significant. Primers (**B**) MHV-3, (**C**) MHV-4, and (**D**) MHV-6 show significantly higher MHV RNA expression compared to controls (n = 6 for control, n = 3 for MHV-1); * *p* ≤ 0.05; ns—not significant.

Uterine tissues from MHV-1-inoculated mice, one year post-infection, showed higher levels of MHV RNA than controls across four primer pairs tested (Figure 10). However, the significance of this increase varied by primer. The MHV-2 primer showed higher MHV RNA levels than those in the control group. However, the difference was not statistically significant (Figure 10A). Conversely, measurements with the MHV-3, MHV-4, and MHV-6 primers showed significantly higher MHV RNA expression in tissues from MHV-1-inoculated mice than in control tissues (Figure 10B–D). These results suggest the presence of MHV-1 RNA in inoculated mouse tissues, indicating successful viral inoculation and the presence of viral RNA. The MHV-3, MHV-4, and MHV-6 primers provide stronger evidence of MHV-1 RNA detection in experimental samples.

## 4. Discussion

MHV-1, a coronavirus inoculated into mice, a well-established experimental model for acute and long COVID, induced notable uterine changes, including fibroid-like lesions. Our study is the first to demonstrate a link between long COVID and fibroid-like lesion formation in murine models. H&E staining of murine uteri after one year of MHV-1 infection revealed chronic inflammation, characterized by interstitial infiltrates of lymphocytes and plasma cells, along with inspissated intraluminal fibrinoid deposits.

Immunofluorescent imaging further demonstrated elevated expression of multiple markers implicated in uterine fibroid pathogenesis, as well as the presence of viral particles distributed throughout the uterine tissue.

Notably, no single virus has been definitively shown to cause human leiomyomas, though some studies suggest that JC virus (JCV) infection may contribute to their pathogenesis. Research on whether Herpes Simplex Virus Type 2 (HSV-2) or other reproductive tract infections affect fibroid development has yielded mixed or inconclusive results, with some studies examining their prevalence in affected women. Some studies have also examined other viruses, such as HPV (human papillomavirus) and EBV (Epstein–Barr virus), in relation to gynecological tumors, but their link to fibroids is not typically considered a primary cause. Since we frequently see these fibroid-like lesions in an animal model of long COVID, there needs to be a serious investigation in humans, not only of COVID but also of other viruses in the future. Additionally, the exact root cause of leiomyomas is unknown, but they are believed to stem from a combination of genetic mutations (e.g., the *med12* gene, found in ~70% of tumors), hormonal influences, and environmental factors. They likely originate from a single stem cell in the uterine muscle tissue, which divides and grows under these influences.

In the uterus, lymphocyte infiltration typically occurs in the cervix, and massive lymphocytic infiltration within uterine fibroids is uncommon [38,39]. However, lymphocytic infiltration of these tumors has been reported in several rare cases [38,40,41]. We observed dense lymphocytic and plasma cell deposits and germinal center formation in murine uterine fibroid-like lesions. These deposits remain within the fibroid-like lesions and do not spread to the myometrium [41]. These lymphocyte deposits often contain infiltrating plasma cells, lymphoblasts, and eosinophils [42]. The cause of this infiltration remains unknown, but it is likely reactive in uterine fibroid-like lesions. Lymphocyte infiltration indicates a strong immune response against tumor growth and is commonly observed in certain tumors, such as melanoma and Hodgkin lymphoma [38,43]. In our murine model, lymphocytes do not undergo rapid division, as in cancer, but are instead recruited to respond to the non-malignant growth of the fibroid-like lesions [38].

We also documented several histopathological findings of chronic inflammation in pathological observations of murine uteri one year after MHV-1 infection. We found intraluminal proteinaceous fluid, an ectatic lumen, and inspissated intraluminal fibrinoid material. These findings likely result from a chronic inflammatory response to abnormal leiomyoma-like myometrial alterations. We recognize that the bicornuate mouse uterus differs anatomically and biologically from the human uterus. Some features observed—such as lymphoplasmacytic infiltrates, intraluminal proteinaceous material, ectatic lumina, and focal hyperplasia—may reflect chronic inflammation, endometritis-like changes, post-inflammatory remodeling, or other uterine pathologies rather than definitive leiomyomas.

A chronic inflammatory state increases vascular permeability, leading to proteinaceous fluid accumulation in the lumen [44]. Inflammation also weakens the blood vessels in the lumen, and increased pressure from fluid accumulation forces them to expand, resulting in an ectatic lumen [45]. This proteinaceous fluid then accumulates in the lumen, where water is reabsorbed, leaving inspissated fibrinoid aggregates [46]. H&E staining further revealed chronic subepithelial and subendometrial inflammation in the uteri of MHV-1-infected mice. The accumulation of uterine fibroids, particularly submucosal fibroids, is associated with an increased prevalence of chronic endometritis [47]. Furthermore, recent findings suggest that this type of chronic inflammation may support leiomyoma growth by triggering the proliferation of clonal myometrial cells and the differentiation of fibroblasts into myofibroblasts [48]. This finding demonstrates a basis for leiomyoma formation during long COVID. Initial chronic inflammation in the uterine lumen triggers a cascade of events leading to fibroid formation, and the accumulation of clonal myocytes further drives subepithelial and subendometrial inflammation, stimulating cell proliferation and supporting leiomyoma growth.

Immunofluorescent imaging of uteri from MHV-1-infected mice revealed upregulated protein expression of several leiomyoma markers. One of these markers, PLP1, is produced by oligodendrocytes and is important for axon myelination [49]. PLP1 is highly upregulated in uterine fibroids, is suspected to promote the growth of these non-malignant tumors and has potential as a diagnostic marker for uterine fibroids [50]. In our MHV-1-infected mice, we observed increased PLP1 expression, further supporting the idea that long COVID may promote uterine fibroid development. We also observed upregulation of versican, an extracellular matrix protein important for regulating cell adhesion, proliferation, and migration [51]. These roles mean that versican can support tumor growth when overexpressed, and it has been shown to support leiomyosarcoma growth in mice [52]. Although the role of versican in leiomyoma pathogenesis has not been elucidated, it likely plays a similar role in supporting the growth of these non-malignant uterine tumors, as both versican isoforms (V0 and V1) are upregulated in women with symptomatic leiomyoma [53]. The enhanced versican expression we observed in our murine models further supports the association between long COVID and uterine fibroid-like lesion development.

The serum biomarker analysis further highlights that tissue protein expression and serum protein concentrations do not necessarily correlate. While immunofluorescence demonstrated increased uterine expression of both PLP1 and versican, only versican showed a slight change in serum, whereas serum PLP1 remained relatively unchanged. This distinction aligns with the biological roles of these proteins. PLP1 is an integral membrane proteolipid that is not a classically secreted protein [54]. Researchers have primarily investigated it as a tissue biomarker of uterine fibroids, with studies showing significant upregulation at both the mRNA and protein levels in leiomyoma tissue [50]. We detected MHV-1 mRNA in uterine tissue from immunocompetent mice infected with MHV-1 one year post-infection (Table 1), although productive replication was not detectable at that time point. MHV-1 is typically an acute respiratory/systemic murine coronavirus infection: infectious virus peaks early and is controlled within days to a few weeks. One-year phenotypes reported after MHV-1—persistent pulmonary, cardiac, or CNS pathology—do not by themselves demonstrate viral persistence or ongoing replication [10]. The important exception is that other neurotropic MHV strains—especially JHM/JHMV and A59—can leave persistent viral RNA and antigen in the mouse CNS for a year or longer, usually in oligodendrocytes and astrocytes, despite failure to recover infectious virus after roughly 10–20 days in immunocompetent animals. That persistence is typically characterized as extremely low-level, immune-controlled, replication-competent viral persistence rather than sustained productive infection. We present Figure 8 because MHV-1 causes acute pneumotropic disease that generally resolves in the lung by day 21 in immunocompetent mice; we are investigating whether uterine positivity occurs later and, if so, whether durable uterine positivity represents a novel, tissue-specific observation of active replication. As noted, this exploratory study reports uterine fibroid-like lesions; we will validate the virus’s presence and its impact on the uterus in future work.

Although PLP1 has shown excellent diagnostic performance in tissue-based analyses, its role as a serum biomarker remains unestablished, and current evidence supporting PLP1 is derived almost exclusively from uterine tissue rather than serum [54,55]. Therefore, the lack of a significant increase in serum PLP1 in the present study suggests that PLP1 may better reflect local molecular changes within the uterus than systemic changes detectable in serum.

In contrast, versican is a major extracellular matrix (ECM) proteoglycan that plays a critical role in regulating matrix organization, cellular proliferation, migration, and tissue remodeling [51,53]. Excessive ECM deposition characterizes uterine fibroids, and human and experimental studies have consistently reported increased versican expression [51,53]. Notably, despite elevated tissue expression, women with uterine fibroids have significantly lower serum versican concentrations than healthy controls, and serum levels show a negative correlation with fibroid volume [56]. This finding closely mirrors the present study, in which uterine versican expression increased while circulating versican decreased after MHV-1 infection. One proposed explanation is that versican becomes increasingly incorporated into and retained within the expanding ECM during fibroid development, thereby reducing the amount of intact protein released into the circulation. In addition, ADAMTS family metalloproteinases continuously remodel versican, generating biologically active cleavage fragments that contribute to ECM turnover and inflammation [53]. Consequently, serum versican concentrations may reflect the balance among local ECM deposition, proteolytic degradation, and systemic clearance rather than total uterine versican abundance [51,53]. These findings emphasize that circulating biomarkers should be interpreted in the context of protein biology and tissue localization. Nevertheless, the concordance between our murine findings and recent human studies supports versican as a promising biomarker of uterine fibroid pathogenesis and suggests that altered serum versican may reflect disease-associated ECM remodeling rather than decreased tissue expression.

Uterine fibroid growth is primarily hormone-driven, as they typically shrink after menopause. The main hormones involved in this growth are estrogen and progesterone, and expression of both receptors is significantly increased in women with uterine fibroids compared with those without [57,58]. However, in our study, we found that high estradiol levels with lower progesterone often reflect a hormonal imbalance called estrogen dominance, which commonly encourages uterine fibroid growth [59,60,61]. Our findings clearly capture a specific intersection of systemic viral stress and local chronic inflammation-driven tissue remodeling or localized hyperactivation of proliferation pathways. Briefly, MHV-1 triggers a severe, acute systemic inflammatory response characterized by a surge in pro-inflammatory cytokines such as IL-1, IL-6, and TNF-alpha. High levels of circulating cytokines and systemic viral stress activate the hypothalamic–pituitary–adrenal (HPA) axis (boosting cortisol/corticosterone) while aggressively shutting down the hypothalamic–pituitary–ovarian (HPO) axis. This suppression may halt the natural estrous cycle in these mice. Because ovulation and functional luteum formation are arrested, systemic progesterone production fails or becomes entirely undetectable. However, even though the ovaries are suppressed systemically, E2 remains present because peripheral tissues synthesize it locally. The massive influx of cytokines (especially IL-6 and TNF-alpha) directly upregulates CYP19A1 (aromatase) expression within reproductive tract tissues.

Aromatase converts circulating adrenal androgens into 17-β estradiol locally within uterine tissue. This allows tissue-level E2 signaling to remain highly active even when systemic ovarian function is minimal. Under these conditions, uterine fibroid-like pathology results directly from estrogen dominance combined with virus-induced tissue repair. Uterine fibroids (leiomyomas) are highly dependent on estrogen for cellular proliferation and extracellular matrix (ECM) deposition. Eliminating progesterone (which usually regulates or halts uncontrolled estrogenic growth in mice) while maintaining E2 creates a state of unmitigated estrogenic drive. Beta coronaviruses are known to induce severe tissue remodeling, fibrin deposition, and fibroproliferative responses. In the uterus, local E2 combined with MHV-1-mediated chronic inflammation may trigger myometrial proliferation and heavy collagen/ECM deposition that histologically resembles a uterine fibroid.

Progesterone receptors exist in two main isoforms, PR-A and PR-B, both of which are overexpressed in uterine fibroids [60,61,62]. Progesterone is believed to influence multiple factors that support uterine fibroid growth, including enhancing TGF-β and IGF-1 expression to promote leiomyoma proliferation and suppressing TNF-α to prevent apoptosis in uterine fibroid cells [60,61,62,63]. TGF-β exists in three isoforms, TGF-β1, TGF-β2, and TGF-β3, and is recognized as a major factor in uterine fibroid pathology. Excessive TGF-β production enhances cellular proliferation and migration to the tumor, thereby stimulating leiomyomas [54,58,64,65]. IGF-1 has also been shown to stimulate uterine fibroid growth by activating PI3K/AKT/mTOR signaling in the uterus [66,67,68,69,70,71,72,73,74,75]. TNF-α mainly inhibits proliferation and induces apoptosis, and its suppression is common in many malignancies, allowing tumors to grow uncontrollably. In progesterone-responsive leiomyomas, TNF-α expression is decreased, particularly under enhanced signaling [63]. We also observed increased expression of the estrogen receptors ER-α and ER-β. ER-α is overexpressed in uterine fibroids; upon binding, estrogen forms an ER-α complex and translocates to the nucleus, where it stimulates transcription of cyclin and growth factor genes [76]. ER-β acts as a negative regulator of ER-α, as it can dimerize with ER-α and prevent it from binding estrogen and altering transcription. Although ER-β may be overexpressed in uterine fibroids, ER-α expression is usually much higher, preventing this negative regulation. In our long COVID mouse models, we observed increased expression of PR-A, PR-B, ER-α, ER-β, TGF-β, IGF-1, and TNF-α, which accurately reflects the hormonal influence on uterine fibroids. These results further support the idea that long COVID may be associated with the development of uterine fibroids.

The serum ELISA findings further support the biological relevance of these signaling pathways and underscore that not all growth factors exhibit similar circulating behavior. Among the proteins evaluated, IGF-1 showed the most pronounced systemic response, with an approximately 2.5-fold increase after MHV-1 infection. Unlike structural proteins such as PLP1 or extracellular matrix proteins such as versican, IGF-1 functions as an endocrine, paracrine, and autocrine growth factor that normally circulates in the bloodstream and acts within target tissues. By activating the IGF-1 receptor, IGF-1 stimulates both the PI3K/AKT/mTOR and Ras/Raf/MAPK signaling pathways, promoting cellular proliferation, protein synthesis, survival, and extracellular matrix production, all of which contribute to leiomyoma growth [69,70,71,72]. Previous studies have shown increased IGF-1 signaling in uterine fibroids, with elevated IGF-1 protein expression correlating with AKT activation and larger tumor size [73]. The significant elevation of circulating IGF-1 observed in the present study therefore suggests that chronic MHV-1 infection may not only induce local uterine IGF-1 signaling but also promote a systemic endocrine environment favorable for fibroid growth.

Although these findings suggest that circulating IGF-1 may be a minimally invasive biomarker of disease progression, current clinical evidence remains limited. IGF-1 is a promising biomarker because of its central role in fibroid biology and in multiple proliferative signaling pathways [66,69,71]. However, circulating IGF-1 is derived predominantly from the liver, and its concentration is influenced by age, growth hormone activity, nutritional status, and other systemic factors. Consequently, serum IGF-1 concentrations should not be interpreted as a specific measure of uterine IGF-1 production or signaling [77]. Thus, serum IGF-1 alone is unlikely to serve as a definitive diagnostic marker but may prove valuable as part of a broader biomarker panel when interpreted alongside imaging findings and other molecular markers.

In contrast, TGF-β1 showed only a modest, nonsignificant increase in serum despite increased uterine expression. This observation is biologically plausible because TGF-β signaling within uterine fibroids is predominantly regulated by local tissue activity rather than by circulating concentrations [58,65]. TGF-β is secreted as an inactive latent complex that remains bound within the extracellular matrix until activated by local proteases, integrins, or mechanical remodeling of the surrounding tissue [78]. Consequently, increased TGF-β1 activity within the uterus does not necessarily translate into proportional increases in serum TGF-β1 concentrations [78]. Once activated, TGF-β stimulates fibroblast proliferation, collagen synthesis, fibronectin production, and proteoglycan deposition, contributing to the excessive extracellular matrix accumulation that characterizes uterine fibroids [58,75]. The modest serum increase observed in the present study may therefore reflect localized activation of profibrotic signaling rather than widespread systemic release of TGF-β1.

Previous clinical studies assessing circulating TGF-β in women with uterine fibroids have yielded variable results, with some reporting significantly increased serum concentrations that correlate with fibroid burden, whereas others suggest that tissue expression more accurately reflects biological activity [58,78]. These discrepancies likely reflect differences in disease stage, sample processing, and the complex regulation of latent versus active TGF-β. Taken together, the present findings suggest that circulating TGF-β1 may be less sensitive than tissue expression for detecting early fibroid-associated molecular changes. Nevertheless, the observed trend toward increased serum TGF-β1 remains consistent with activation of profibrotic pathways and warrants further investigation in larger longitudinal studies to evaluate its utility as part of a multimarker panel for uterine fibroid development.

MSH2 encodes the MSH2 protein, which is vital for DNA mismatch repair [79,80]. We observed increased levels of MSH2 protein but decreased levels of MSH2 mRNA. This apparent contradiction is consistent with previous findings on uterine fibroids. Enhancer of Zeste Homolog 2 (EZH2) epigenetically inhibits MSH2 expression by depositing the histone mark H3K27me3, while a separate compensatory mechanism is triggered to increase the translation and stability of the existing MSH2 proteins. This upregulation of MSH2 is common in uterine fibroids and may serve as a marker for early detection [80]. Dysregulated DNA mismatch repair is believed to be one of the drivers of uterine fibroid formation, and the downregulation of MSH2 is a likely explanation for the faulty DNA repair and frequent genetic mutations seen in uterine fibroid tissue [79,80]. Our findings mirror what is known about leiomyoma pathogenesis and further support the possibility that long COVID can induce uterine fibroids.

CA125 is often elevated in women with uterine fibroids and can serve as a nonspecific blood biomarker [81]. CA125 levels correlate with fibroid size and the presence of adenomyosis [82]. Although CA125 does not have a clear role in leiomyoma pathogenesis, the elevated levels observed in our study suggest tumorigenesis and are similar to those seen in women with uterine fibroids. HMGA2 is a gene active during fetal development that is typically inactive in adult tissues [82]. However, this gene can be reactivated under certain conditions, including leiomyoma. HMGA2 overexpression is common in uterine leiomyomas and is often used as a diagnostic biomarker [83]. This overexpression enhances cellular proliferation and fibroid growth, and, in different cell types, it can induce leiomyoma-like conditions [84]. We observed HMGA2 overexpression in our study, further indicating uterine fibroid-like lesion formation in our long COVID model mice.

CEACAM1 is a tumor-suppressing protein that is greatly downregulated in uterine fibroids compared to healthy tissue [85]. Downregulation of CEACAM1 mRNA and its protein enhances cellular proliferation and leads to fibroid growth. We observed the same decrease in CEACAM1 in the uteri of our mice 1 year after MHV-1 infection, suggesting that long COVID may enhance uterine fibroid development. Further, infection-induced receptor downregulation, rather than receptor loss, may increase viral RNA and reduce susceptibility to subsequent entry without stopping the infection already present. For example, MHV-1 proteins downregulate CD4 and coreceptors during productive infection.

Most differentially expressed genes in the SPK-treated group were restored to baseline levels, similar to the control. Serum biomarker responses were more variable than tissue findings, with some proteins trending toward normalization while others remained largely unchanged. These findings are not unexpected, as serum protein concentrations are influenced by multiple factors, including protein synthesis, secretion, ECM retention, and systemic clearance. This discordance indicates that serum protein markers may not immediately reflect therapeutic changes within the uterus and warrants further investigation in larger longitudinal studies.

## 5. Conclusions

**Figure 11:**
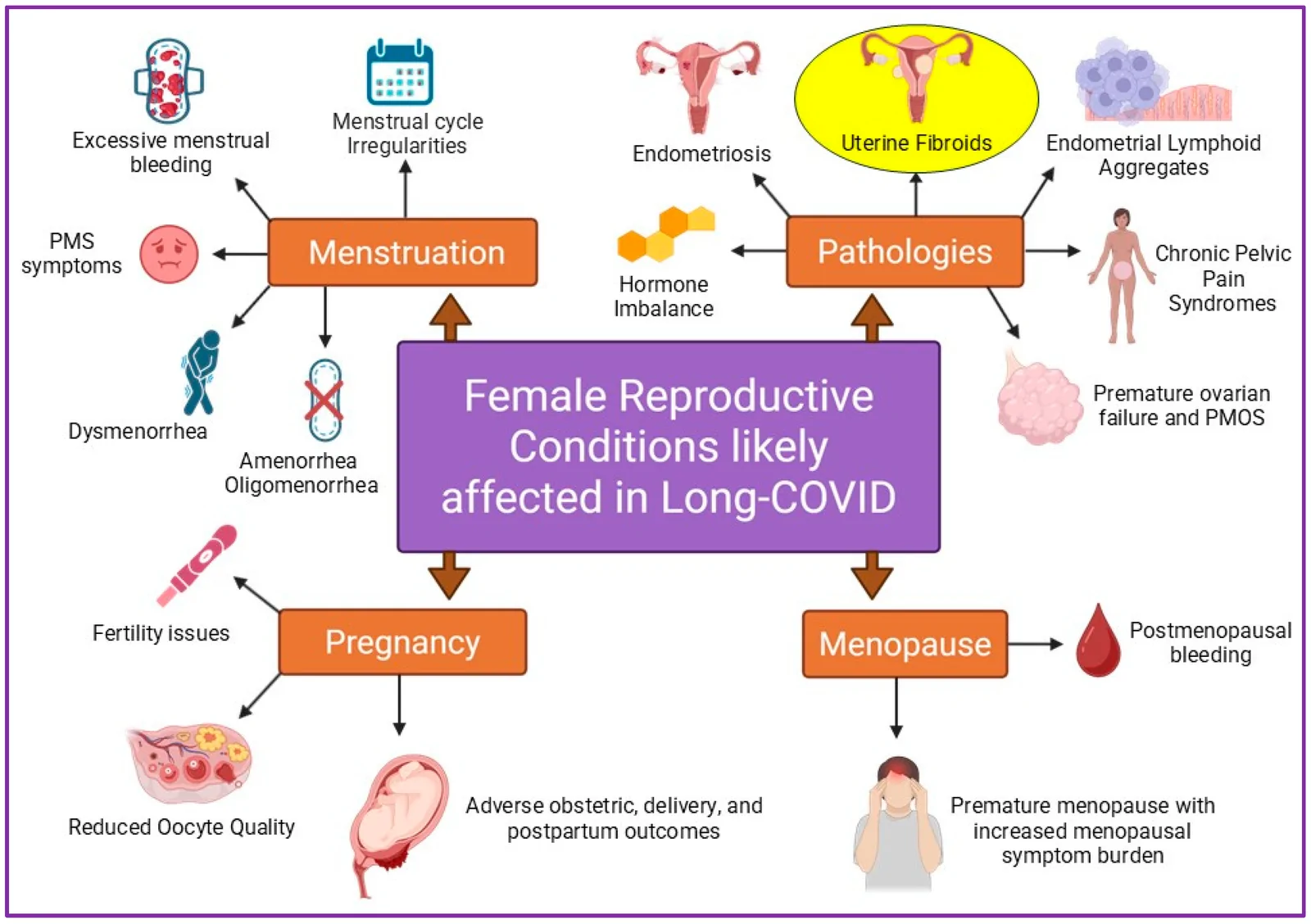
Reproductive symptoms and conditions that may be associated with long COVID.

Although COVID-19 infection and vaccination have been linked to temporary menstrual irregularities, including heavier bleeding, prolonged cycles, and spotting between periods, the exact nature of COVID-19’s impact on reproductive health, particularly in long COVID, remains poorly studied (Figure 11).

This pilot study identifies chronic uterine inflammatory and fibroid-like histopathological changes, altered immunoreactivity of fibroid-associated protein markers, and elevated serum IGF-1 and 17-beta estradiol in a long-term MHV-1 mouse model. These findings do not definitively establish the presence of leiomyomas, active uterine viral replication, a causal role for MHV-1 in lesion formation, or a direct link between fibroid development and long COVID. Thus, larger, blinded, longitudinal studies in human validation cohorts are required.

## Figures and Tables

**Table 1:** Primers used in the RT-PCR analysis.

Primer	Sequence (5’-3’)
M-MHV-F1/R1	CGTTGGGCATTATACTACTCTTTATTACTATC/CAAAATACATAATCCACATTACAATGGACAC
M-MHV-F2/R2	CTACTCTTTATTACTATCATACTACAGTTCGG/GTCCTGATAAACAACCTAATGCTATTAACAAA
M-MHV-F3/R3	TACTCTTTATTACTATCATACTACAGTTCGGT/CAAAATACATAATCCACATTACAATGGACACT
M-MHV F4/R4	CATTATACTACTCTTTATTACTATCAT/TGCTATTAACAAAATACATAATCCA
M-MHV-F5/R5	CATTATACTACTCTTTATTACTATCATACT/AATGCTATTAACAAAATACATAATCCA
M-MHV-F6/R6	CATTATACTACTCTTTATTACTATCATACT/CAAAATACATAATCCACATTACAAT

## Data Availability

The data presented in this study are available upon request from the corresponding author. However, due to the University of Miami Miller School of Medicine’s privacy policy, they are not publicly available.

## Abbreviations

The following abbreviations are used in this manuscript:

ACE2	Angiotensin-Converting Enzyme 2
ANOVA	Analysis of Variance
AUB	Abnormal Uterine Bleeding
CEACAM1	Carcinoembryonic antigen-related cell adhesion molecule 1
COVID	Coronavirus Disease
EBV	Epstein–Barr virus
ECM	extracellular matrix
ELISA	Enzyme-Linked Immunosorbent Assay
FIGO	International Federation of Gynecology and Obstetrics
HMGA2	high-mobility group AT-hook 2
HPV	human papillomavirus
HPV	Herpes Simplex Virus Type 2
IGF-1	insulin-like growth factor-1
IHC	Immunohistochemistry
JCV	John Cunningham (Human polyomavirus)
LD	Linear Dichroism
ME-CFS	Myalgic encephalomyelitis-chronic fatigue syndrome
MED12	Mediator Complex Subunit 12
MHV-1	Murine Hepatitis virus
MSH2	MutS homolog 2
PLP1	proteolipid protein 1
PR-A	Progesterone Receptor A
PR-B	Progesterone Receptor B
SARS-CoV-2	severe acute respiratory syndrome coronavirus-2
SPK	Spikenet
TGFβ	Transforming Growth Factor Beta
TNFα	Tumor Necrosis Factor Alpha

## References

[1] Ayoub H.H., Chemaitelly H., Abu-Raddad L.J. (2025). Global Estimates of COVID-19 Morbidity and Mortality: A Cohort Study and Mathematical Model Analysis. Influenza Other Respir. Viruses.

[2] World Health Organization (2025). Coronavirus Disease (COVID-19).

[3] Al-Aly Z., Davis H., McCorkell L., Soares L., Wulf-Hanson S., Iwasaki A., Topol E.J. (2024). Long COVID science, research and policy. Nat. Med..

[4] Mortaz E., Tabarsi P., Jamaati H., Dalil Roofchayee N., Dezfuli N.K., Hashemian S.M., Moniri A., Marjani M., Malekmohammad M., Mansouri D. (2021). Increased Serum Levels of Soluble TNF-α Receptor Is Associated with ICU Mortality in COVID-19 Patients. Front. Immunol..

[5] Li Y., Tenchov R., Smoot J., Liu C., Watkins S., Zhou Q. (2021). A Comprehensive Review of the Global Efforts on COVID-19 Vaccine Development. ACS Cent. Sci..

[6] Al-Hatamleh M.A.I., Abusalah M.A., Hatmal M.M., Alshaer W., Ahmad S., Mohd-Zahid M.H., Rahman E.N.S.E.A., Yean C.Y., Alias I.Z., Uskoković V. (2023). Understanding the challenges to COVID-19 vaccines and treatment options, herd immunity and probability of reinfection. J. Taibah Univ. Med. Sci..

[7] Korrner R.W., Majjouti M., Alcazar M.A.A., Mahabir E. (2020). Of Mice and Men: The Coronavirus MHV and Mouse Models as a Translational Approach to Understand SARS-CoV-2. Viruses.

[8] De Albuquerque N., Baig E., Ma X., Zhang J., He W., Rowe A., Habal M., Liu M., Shalev I., Downey G.P. (2006). Murine hepatitis virus strain 1 produces a clinically relevant model of severe acute respiratory syndrome in A/J mice. J. Virol..

[9] Gong H.H., Worley M.J., Carver K.A., Goldstein D.R., Deng J.C. (2023). Neutrophils drive pulmonary vascular leakage in MHV-1 infection of susceptible A/J mice. Front. Immunol..

[10] Paidas M.J., Cosio D.S., Ali S., Kenyon N.S., Jayakumar A.R. (2022). Long-Term Sequelae of COVID-19 in Experimental Mice. Mol. Neurobiol..

[11] Paidas M.J., Sampath N., Schindler E.A., Cosio D.S., Ndubizu C.O., Shamaladevi N., Kwal J., Rodriguez S., Ahmad A., Kenyon N.S. (2022). Mechanism of Multi-Organ Injury in Experimental COVID-19 and Its Inhibition by a Small Molecule Peptide. Front. Pharmacol..

[12] Caldera-Crespo L.A., Paidas M.J., Roy S., Schulman C.I., Kenyon N.S., Daunert S., Jayakumar A.R. (2022). Experimental Models of COVID-19. Front. Cell. Infect. Microbiol..

[13] Ramamoorthy R., Hussain H., Ravelo N., Sriramajayam K., Di Gregorio D.M., Paulrasu K., Chen P., Young K., Masciarella A.D., Jayakumar A.R. (2023). Kidney Damage in Long COVID: Studies in Experimental Mice. Biology.

[14] Paidas M.J., Mohamed A.B., Norenberg M.D., Saad A., Barry A.F., Colon C., Kenyon N.S., Jayakumar A.R. (2021). Multi-Organ Histopathological Changes in a Mouse Hepatitis Virus Model of COVID-19. Viruses.

[15] Masciarella A.D., Di Gregorio D.M., Ramamoorthy R., Hussain H., Jayakumar A.R., Paidas M.J. (2023). A Mouse Model of MHV-1 Virus Infection for Study of Acute and Long COVID Infection. Curr. Protoc..

[16] Ramamoorthy R., Speciale A.R., West E.M., Hussain H., Elumalai N., Schmitz Abe K.E., Paidas M.J., Jayakumar A.R. (2025). Persistent Inflammation, Maladaptive Remodeling, and Fibrosis in the Kidney Following Long COVID-like MHV-1 Mouse Model. Diseases.

[17] Cox G., Gonzalez A.J., Ijezie E.C., Rodriguez A., Miller C.R., Van Leuven J.T., Miura T.A. (2022). Priming with Rhinovirus Protects Mice Against a Lethal Pulmonary Coronavirus Infection. Front. Immunol..

[18] Gain C., Song S., Angtuaco T., Satta S., Kelesidis T. (2023). The role of oxidative stress in the pathogenesis of infections with coronaviruses. Front. Microbiol..

[19] Somasundaram N.P., Ranathunga I., Ratnasamy V., Wijewickrama P.S.A., Dissanayake H.A., Yogendranathan N., Gamage K.K.K., de Silva N.L., Sumanatilleke M., Katulanda P. (2020). The Impact of SARS-CoV-2 Virus Infection on the Endocrine System. J. Endocr. Soc..

[20] Todorova M., Tsvetkova V., Atanasova M., Ruseva A., Todorova K. (2026). Glucocorticoid Resistance in COVID-19: Endocrine–Inflammatory Associations in a Cross-Sectional Study. COVID.

[21] Benny M., Bandstra E.S., Saad A.G., Lopez-Alberola R., Saigal G., Paidas M.J., Jayakumar A.R., Duara S. (2023). Maternal SARS-CoV-2, Placental Changes and Brain Injury in 2 Neonates. Pediatrics.

[22] Prakash S., Karan S., Lekbach Y., Tifrea D.F., Figueroa C.J., Ulmer J.B., Young J.F., Glenn G., Gil D., Jones T.M. (2025). Insights into Persistent SARS-CoV-2 Reservoirs in Chronic Long COVID. Viruses.

[23] National Academies of Sciences, Engineering, and Medicine (2024). Long-Term Health Effects of COVID-19: Disability and Function Following SARS-CoV-2 Infection.

[24] Rahimzadeh H., Kazemian S., Rahbar M., Farrokhpour H., Montazeri M., Kafan S., Salimzadeh A., Talebpour M., Majidi F., Jannatalipour A. (2021). The Risk Factors and Clinical Outcomes Associated with Acute Kidney Injury in Patients with COVID-19: Data from a Large Cohort in Iran. Kidney Blood Press. Res..

[25] Maybin J.A., Walker C., Watters M., Homer N.Z., Simpson J.P., Robb C., Gibson D.A., Jeanjean L., Critchley H.O.D., Kountourides G. (2025). The potential bidirectional relationship between long COVID and menstruation. Nat. Commun..

[26] Madaan S., Talwar D., Jaiswal A., Kumar S., Acharya N., Acharya S., Dewani D. (2022). Post-COVID-19 menstrual abnormalities and infertility: Repercussions of the pandemic. J. Educ. Health Promot..

[27] Maham S., Yoon M.S. (2024). Clinical Spectrum of Long COVID: Effects on Female Reproductive Health. Viruses.

[28] Moustakli E., Stavros S., Michaelidis T.M., Potiris A., Christodoulaki C., Zachariou A., Drakakis P., Zikopoulos K., Domali E., Zikopoulos A. (2025). Long-Term Effects of COVID-19 on Women’s Reproductive Health and Its Association with Autoimmune Diseases, Including Multiple Sclerosis. J. Clin. Med..

[29] Starek E., Żak K., Ostrowska-Leśko M., Bobiński M. (2026). The Impact of Long COVID on the Female Reproductive System: A Narrative Review. Int. J. Mol. Sci..

[30] Bulun S.E., Yin P., Wei J., Zuberi A., Iizuka T., Suzuki T., Saini P., Goad J., Parker J.B., Adli M. (2025). UTERINE FIBROIDS. Physiol. Rev..

[31] Barjon K., Kahn J., Singh M. (2026). Uterine Leiomyomata. StatPearls.

[32] Stewart E.A., Laughlin-Tommaso S.K. (2024). Uterine Fibroids. N. Engl. J. Med..

[33] Uimari O., Subramaniam K.S., Vollenhoven B., Tapmeier T.T. (2022). Uterine Fibroids (Leiomyomata) and Heavy Menstrual Bleeding. Front. Reprod. Health.

[34] Ghant M.S., Sengoba K.S., Vogelzang R., Lawson A.K., Marsh E.E. (2016). An Altered Perception of Normal: Understanding Causes for Treatment Delay in Women with Symptomatic Uterine Fibroids. J. Womens Health.

[35] Ali M., Ciebiera M., Vafaei S., Alkhrait S., Chen H.Y., Chiang Y.F., Huang K.C., Feduniw S., Hsia S.M., Al-Hendy A. (2023). Progesterone Signaling and Uterine Fibroid Pathogenesis; Molecular Mechanisms and Potential Therapeutics. Cells.

[36] Rick F.G., Abi-Chaker A., Szalontay L., Perez R., Jaszberenyi M., Jayakumar A.R., Shamaladevi N., Szepeshazi K., Vidaurre I., Halmos G. (2013). Shrinkage of experimental benign prostatic hyperplasia and reduction of prostatic cell volume by a gastrin-releasing peptide antagonist. Proc. Natl. Acad. Sci. USA.

[37] Wang X., Sui X., Ma Y., Li M., Zhang X., Fei D., Ma M. (2022). Real-time reverse transcription recombinase polymerase amplification for rapid detection of murine hepatitis virus. Front Microbiol..

[38] Prete M.D., Collamarini M., Collamarini C., Sesti F., Mauriello A., Palmieri G. (2020). Uterine Leiomyoma with Massive Lymphoid Infiltrate in a Patient with History of Assisted In-Vitro Fertilization. Turk. Patoloji Derg..

[39] Young R.H., Harris N.L., Scully R.E. (1985). Lymphoma-like lesions of the lower female genital tract: A report of 16 cases. Int. J. Gynecol. Pathol..

[40] Wasyluk T., Obrzut B., Gałązka K., Żmuda M., Obrzut M., Darmochwał-Kolarz D. (2019). Uterine myoma with massive lymphocytic infiltration—Case report. Przegl. Menopauzalny.

[41] Provencher D., Wong J., Rahimi K., Maietta A., Roy S.F. (2022). Leiomyoma with Massive Lymphoid Infiltration: 2 Cases with Lymphocytic Vasculopathy and Angiocentric Germinal Centers. Int. J. Gynecol. Pathol..

[42] Ferry J.A., Harris N.L., Scully R.E. (1989). Uterine leiomyomas with lymphoid infiltration simulating lymphoma. A report of seven cases. Int. J. Gynecol. Pathol..

[43] Bida M., Miya T.V., Hull R., Dlamini Z. (2024). Tumor-infiltrating lymphocytes in melanoma: From prognostic assessment to therapeutic applications. Front. Immunol..

[44] Wiig H. (2011). Pathophysiology of tissue fluid accumulation in inflammation. J. Physiol..

[45] Al-Zakhari R., Aljammali S., Galligan S., Rotatori F. (2021). Coronary Artery Ectasia: A Case Report Discussing the Causes, Diagnosis, and Treatment. Cureus.

[46] Adolph R., Vorp D.A., Steed D.L., Webster M.W., Kameneva M.V., Watkins S.C. (1997). Cellular content and permeability of intraluminal thrombus in abdominal aortic aneurysm. J. Vasc. Surg..

[47] Ghanaei M.M., Sharami S.H., Heirati S.F.D., Etezadi A., Pourhabibi Z., Hosseinzadeh F., Kabodmehri R. (2022). The association between chronic endometritis and uterine fibroids. J. Fam. Med. Prim. Care.

[48] Rakhmatullaeva M.M., Khaydarova F.A. (2025). Inflammation as a Factor Initiating the Growth of Uterine Fibroids. Am. J. Med. Med. Sci..

[49] Wolf N.I., van Spaendonk R.M.L., Hobson G.M., Adam M.P., Bick S., Mirzaa G.M. (1993–2026). PLP1-Related Disorders. GeneReviews®.

[50] Cai L., Liao Z., Li S., Wu R., Li J., Ren F., Zhang H. (2022). PLP1 may serve as a potential diagnostic biomarker of uterine fibroids. Front. Genet..

[51] Wight T.N. (2002). Versican: A versatile extracellular matrix proteoglycan in cell biology. Curr. Opin. Cell Biol..

[52] Keire P.A., Bressler S.L., Lemire J.M., Edris B., Rubin B.P., Rahmani M., McManus B.M., van de Rijn M., Wight T.N. (2014). A role for versican in the development of leiomyosarcoma. J. Biol. Chem..

[53] Gueye N.A., Mead T.J., Koch C.D., Biscotti C.V., Falcone T., Apte S.S. (2017). Versican Proteolysis by ADAMTS Proteases and Its Influence on Sex Steroid Receptor Expression in Uterine Leiomyoma. J. Clin. Endocrinol. Metab..

[54] Baron W., Ozgen H., Klunder B., de Jonge J.C., Nomden A., Plat A., Trifilieff E., de Vries H., Hoekstra D. (2015). The major myelin-resident protein PLP is transported to myelin membranes via a transcytotic mechanism: Involvement of sulfatide. Mol. Cell. Biol..

[55] Tsibris J.C., Segars J., Coppola D., Mane S., Wilbanks G.D., O’brien W.F., Spellacy W.N. (2002). Insights from gene arrays on the development and growth regulation of uterine leiomyomata. Fertil. Steril..

[56] Jasti P., Kumari S., Singh S., Anudeep P.P. (2023). Serum versican as a potential biomarker in patients with uterine fibroids: A study from Eastern India. J. Fam. Med. Prim. Care.

[57] Islam M.S., Afrin S., Jones S.I., Segars J. (2020). Selective Progesterone Receptor Modulators-Mechanisms and Therapeutic Utility. Endocr. Rev..

[58] Ciebiera M., Włodarczyk M., Wrzosek M., Męczekalski B., Nowicka G., Łukaszuk K., Ciebiera M., Słabuszewska-Jóźwiak A., Jakiel G. (2017). Role of Transforming Growth Factor β in Uterine Fibroid Biology. Int. J. Mol. Sci..

[59] Moravek M.B., Bulun S.E. (2015). Endocrinology of uterine fibroids: Steroid hormones, stem cells, and genetic contribution. Curr. Opin. Obstet. Gynecol..

[60] Wu X., Wang H., Englund K., Blanck A., Lindblom B., Sahlin L. (2002). Expression of progesterone receptors A and B and insulin-like growth factor-I in human myometrium and fibroids after treatment with a gonadotropin-releasing hormone analogue. Fertil. Steril..

[61] Ishikawa H., Ishi K., Serna V.A., Kakazu R., Bulun S.E., Kurita T. (2010). Progesterone is essential for maintenance and growth of uterine leiomyoma. Endocrinology.

[62] Wang S., You W., Ding D., Liu F., Han F., Tang L. (2026). The estrogen-progestogen-oxidative stress network in uterine fibroids: Mechanistic insights and therapeutic opportunities. Redox Rep..

[63] Kurachi O., Matsuo H., Samoto T., Maruo T. (2001). Tumor necrosis factor-alpha expression in human uterine leiomyoma and its down-regulation by progesterone. J. Clin. Endocrinol. Metab..

[64] Dou Q., Zhao Y., Tarnuzzer R.W., Rong H., Williams R.S., Schultz G.S., Chegini N. (1996). Suppression of transforming growth factor-beta (TGF beta) and TGF beta receptor messenger ribonucleic acid and protein expression in leiomyomata in women receiving gonadotropin-releasing hormone agonist therapy. J. Clin. Endocrinol. Metab..

[65] Lee B.S., Nowak R.A. (2001). Human leiomyoma smooth muscle cells show increased expression of transforming growth factor-beta 3 (TGF beta 3) and altered responses to the antiproliferative effects of TGF beta. J. Clin. Endocrinol. Metab..

[66] Wang C., Sun Y., Cong S., Zhang F. (2023). Insulin-Like Growth Factor-1 Promotes Human Uterine Leiomyoma Cell Proliferation via PI3K/AKT/mTOR Pathway. Cells Tissues Organs.

[67] Yakar S., Wu Y., Setser J., Rosen C.J. (2002). The role of circulating IGF-I: Lessons from human and animal models. Endocrine.

[68] Phillips L.S., Harp J.B., Goldstein S., Klein J., Pao C.I. (1990). Regulation and action of insulin-like growth factors at the cellular level. Proc. Nutr. Soc..

[69] Yu L., Saile K., Swartz C.D., He H., Zheng X., Kissling G.E., Di X., Lucas S., Robboy S.J., Dixon D. (2008). Differential expression of receptor tyrosine kinases (RTKs) and IGF-I pathway activation in human uterine leiomyomas. Mol. Med..

[70] Peng L., Wen Y., Han Y., Wei A., Shi G., Mizuguchi M., Lee P., Hernando E., Mittal K., Wei J.-J. (2009). Expression of insulin-like growth factors (IGFs) and IGF signaling: Molecular complexity in uterine leiomyomas. Fertil. Steril..

[71] Gkioka E., Msaouel P., Philippou A., Vlachogiannis N.I., Vogkou C.T., Margiolis A., Koutsilieris M. (2015). Review: The Role of Insulin-like Growth Factor-1 Signaling Pathways in Uterine Leiomyoma. In Vivo.

[72] LeRoith D., Roberts C.T. (1993). Insulin-like growth factors. Ann. N. Y. Acad. Sci..

[73] Baird D.D., Travlos G., Wilson R., Dunson D.B., Hill M.C., D’Aloisio A.A., London S.J., Schectman J.M. (2009). Uterine leiomyomata in relation to insulin-like growth factor-I, insulin, and diabetes. Epidemiology.

[74] Sozen I., Arici A. (2002). Interactions of cytokines, growth factors, and the extracellular matrix in the cellular biology of uterine leiomyomata. Fertil. Steril..

[75] Islam M.S., Ciavattini A., Petraglia F., Castellucci M., Ciarmela P. (2018). Extracellular matrix in uterine leiomyoma pathogenesis: A potential target for future therapeutics. Hum. Reprod. Update.

[76] Borahay M.A., Asoglu M.R., Mas A., Adam S., Kilic G.S., Al-Hendy A. (2017). Estrogen receptors and signaling in fibroids: Role in pathobiology and therapeutic implications. Reprod. Sci..

[77] Clemmons D.R. (2006). Clinical utility of measurements of insulin-like growth factor 1. Nat. Clin. Pract. Endocrinol. Metab..

[78] Robertson I.B., Rifkin D.B. (2016). Regulation of the bioavailability of TGF-β and TGF-β-related proteins. Cold Spring Harb. Perspect. Biol..

[79] Lin D.P., Wang Y., Scherer S.J., Clark A.B., Yang K., Avdievich E., Jin B., Werling U., Parris T., Kurihara N. (2004). An Msh2 point mutation uncouples DNA mismatch repair and apoptosis. Cancer Res..

[80] Yang Q., Laknaur A., Elam L., Ismail N., Gavrilova-Jordan L., Lue J., Diamond M.P., Al-Hendy A. (2016). Identification of Polycomb Group Protein EZH2-Mediated DNA Mismatch Repair Gene MSH2 in Human Uterine Fibroids. Reprod. Sci..

[81] Bischof P., Galfetti M.A., Seydoux J., von Hospenthal J.U., Campana A. (1992). Peripheral CA 125 levels in patients with uterine fibroids. Hum. Reprod..

[82] Babacan A., Kizilaslan C., Gun I., Muhcu M., Mungen E., Atay V. (2014). CA 125 and other tumor markers in uterine leiomyomas and their association with lesion characteristics. Int. J. Clin. Exp. Med..

[83] Mäkinen N., Kämpjärvi K., Frizzell N., Bützow R., Vahteristo P. (2017). Characterization of MED12, HMGA2, and FH alterations reveals molecular variability in uterine smooth muscle tumors. Mol. Cancer.

[84] Mas A., Cervelló I., Fernández-Álvarez A., Faus A., Díaz A., Burgués O., Casado M., Simón C. (2015). Overexpression of the truncated form of High Mobility Group A proteins (HMGA2) in human myometrial cells induces leiomyoma-like tissue formation. Mol. Hum. Reprod..

[85] DeAngelis A.M., Malik M., Britten J., Driggers P., Catherino W.H. (2021). Carcinoembryonic antigen-related cell adhesion molecule 1: A key regulatory protein involved in leiomyoma growth. F&S Sci..

